# Development of bright NIR-emitting pressure-sensitive paints using benzoporphyrin luminophores†

**DOI:** 10.1039/d5sc00810g

**Published:** 2025-03-20

**Authors:** Elliott J. Nunn, Dimitrios Tsioumanis, Tom B. Fisher, David A. Roberts, Mark K. Quinn, Louise S. Natrajan

**Affiliations:** a Department of Chemistry, University of Manchester Oxford Road M13 9PL UK elliott.nunn@manchester.ac.uk louise.natrajan@manchester.ac.uk; b School of Engineering, University of Manchester Oxford Road M13 9PL UK mark.quinn@manchester.ac.uk; c BAE Systems, Warton Aerodrome Warton PR4 1AX UK; d Aircraft Research Association Manton Lane Bedford MK41 7PF UK

## Abstract

Pressure-sensitive paints (PSPs) are an optical surface pressure sensor for aerodynamic measurements that operates through the oxygen dependent luminescence of a luminophore molecule. The luminophore has remained relatively consistent over the past 20 years, with platinum(ii)/palladium(ii)-5,10,15,20-tetrakis-(2,3,4,5,6-pentafluorphenyl)-porphyrin (Pt/PdTFPP) being popular choices due to their well-known photostability. In this work, NIR-emitting Pt(ii) and Pd(ii) complexes of tetraphenyl tetrabenzoporphyrins and new *para* CF_3_ substituted tetraphenyl tetrabenzoporphyrins have been investigated as improved luminophores in PSP formulations for the first time. The red shifted NIR emission spectra of the benzoporphyrins offer a wider and more conveniently placed spectral window than Pt/PdTFPP, creating more of a spectral gap for a secondary temperature-sensitive luminophore to be used in future binary PSPs. The *para* CF_3_ substituted Pt(ii) and Pd(ii) benzoporphyrins exhibited substantially increased luminescent brightness over PtTFPP and PdTFPP (5× higher), resulting in signficantly brighter PSP formulations. The benzoporphyrins greatly improved the performance of polystyrene based-PSPs, increasing pressure sensitivity by 20% and decreasing temperature sensitivity by 50%, compared to the current gold standard PtTFPP and PdTFPP.

## Abstract 2

Pressure-sensitive paints (PSPs) are an optical surface pressure sensor for aerodynamic measurements that operates through the oxygen dependent luminescence of a luminophore molecule. NIR-emitting Pt(ii) and Pd(ii) benzoporphyrins offer distinct advantages over traditional Pt/PdTFPP based PSPs.

## Graphical abstract

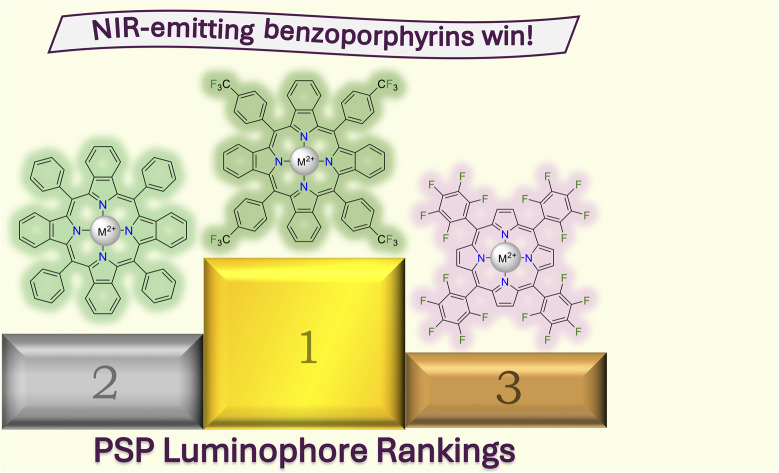

## Introduction

Singlet oxygen photosensitisers have found widespread use in a variety of fields, for example: photodynamic therapy (PDT),^1,2^ biomedical imaging,^3^ chemical synthesis,^4,5^ water treatment^6,7^ and optical oxygen sensors.^8,9^ A notable and unique application of optical oxygen sensors is pressure-sensitive paints (PSPs), which are a powerful tool for measuring full-field surface pressure distributions and visualising aerodynamic phenomena in model-based wind tunnel testing. PSPs are formulations of a photoactive molecule, known as a luminophore, and an oxygen permeable binder which are applied to a model's surface.^10^ Upon illumination the luminophore molecule is excited into the triplet (T_1_) state, which is long enough lived (typically μs lifetime), to be quenched through collisional quenching by dissolved O_2_ in the binder matrix. The luminophore emission can be imaged during wind tunnel operation, and full-field surface pressure maps of the test model, in a given flow, can be generated.^11–14^ PSPs are relatively easy to apply (*via* spraying) and have distinct advantages over traditional pressure taps, which are expensive to implement and only provide low-resolution spatial measurements.

Despite the myriad benefits that PSPs provide, several key features limit their widespread industrial use. A crucial limitation is the inherent temperature sensitivity associated with PSP measurements, which is due to two components: the temperature-dependency of the non-radiative decay of the luminophore and of oxygen diffusion through the binder matrix.^10,15^ Models in wind tunnel experiments often experience large surface temperature gradients and thus this temperature sensitivity can result in inaccurate surface pressure sensing.

Binary PSP formulations attempt to solve this problem, by introducing a secondary luminophore that is normally temperature-sensitive but pressure-insensitive.^16–19^ However, many of these formulations suffer from significant spectral cross-talk and overlap between the two luminophores, causing major performance loss.^16,20^ Many PSP formulations also suffer from low brightness, especially at higher pressures, where the luminescent signal can become challenging to measure, rendering accurate pressure determination difficult, due to the decreased signal-to-noise ratio (SNR).

There has been extensive development on the binder and data acquisition methods for PSPs, which have led to significant improvements in the technology;^20–25^ however, the chemistry of the pressure-sensitive luminophore has been left curiously undeveloped since the early 2000s. In terms of porphyrin-based PSPs, platinum(ii) octaethylporphyrin (PtOEP) was first used due to its large quantum yield.^12^ However, PtOEP is very sensitive to concentration dependant self-quenching at higher dye loadings and suffers from severe photodegradation over prolonged exposure.^26^ Therefore, platinum(ii)-*meso*-tetra(pentafluorophenyl) porphyrin (PtTFPP) quickly replaced PtEOP due to its greater photostability and lower sensitivity to concentration quenching.^27^PtTFPP has since become the predominant luminophore in the majority of PSP formulations.

For binary PSP formulations, the broad red emission of PtTFPP (roughly 650 nm), causes significant spectral overlap with many desirable secondary luminophores, reducing overall performance. Khalil *et al.* synthesised and successfully used platinum(ii)-*meso*-tetra(pentafluorophenyl)porpholactone (PtTFPL), in a binary PSP formulation.^17,28^ PtTFPL possessed a red shifted emission band (733 nm), compared to PtTFPP making it the first NIR-emitting PSP. However, PtTFPL still suffers from a low overall brightness, especially at high pressures.

**Fig. 1:**
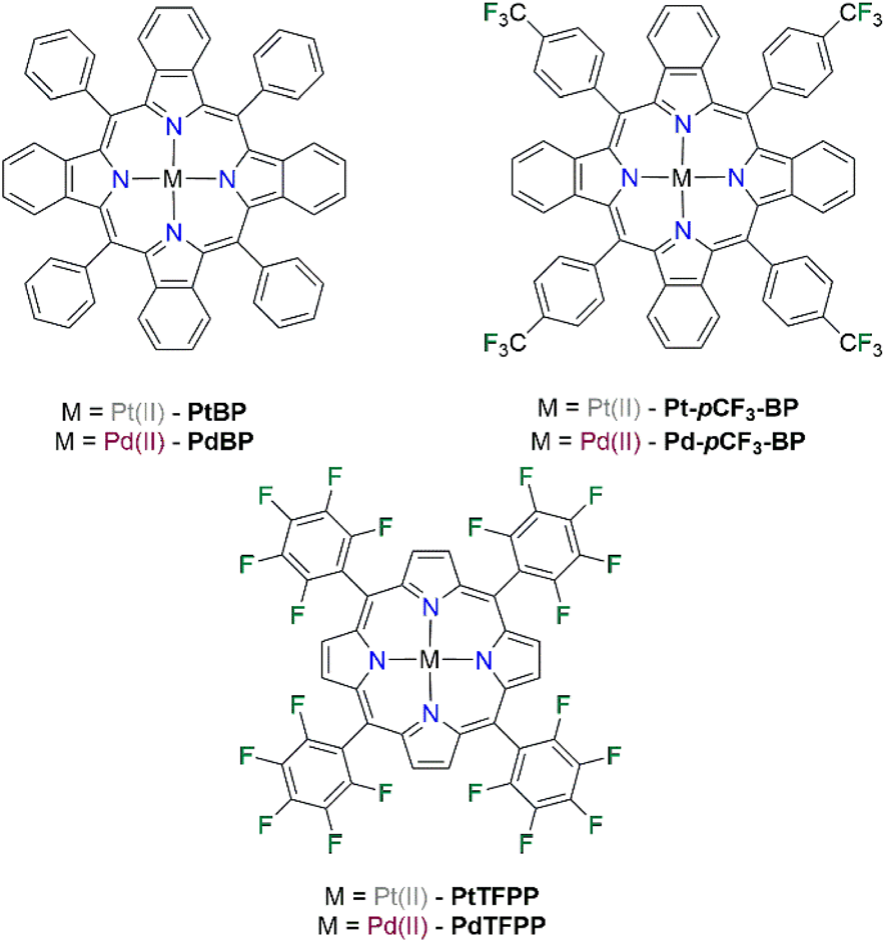
The chemical structures of the Pt(ii) and Pd(ii) benzoporphyrin luminophores used in this study. PtTFPP and PdTFPP are the standard PSP luminophores and are the reference compounds in this study.

In this work, we aim to develop new and improved NIR-emitting PSPs, with the goal of making a brighter PSP that has a larger spectral window for insertion of secondary luminophores to avoid spectral overlap and cross talk in binary formulations. The NIR-emitting luminophores investigated in this work are Pt(ii) and Pd(ii) benzoporphyrins. Benzoporphyrins have found great success in other oxygen-sensing platforms due to their large quantum yields and oxygen sensitivities.^29–31^ We have synthesised Pt(ii) and Pd(ii) tetraphenyl tetrabenzoporphyrin, called PtBP and PdBP in this study, as well as the novel para CF_3_ substituted derivatives denoted as Pt-pCF3-BP and Pd-pCF3-BP (Fig. 1). These benzoporphyrins will then be benchmarked against PtTFPP and PdTFPP (the standard PSP luminophores), to investigate their efficacy as luminophores in new NIR-emitting polymer-based PSPs.

## Results and discussion

### Synthesis

The reference luminophores PtTFPP and PdTFPP were synthesised previously.^32,33^ The unsubstituted and *para* CF_3_ substituted benzoporphyrins were synthesised using the multi-step synthesis developed by Finikova *et al.* (see ESI† for full details).^34^ This synthesis involved the formation of tetrahydroisoindole, and the subsequent Lindsey reaction with benzaldehyde/4-trifluoromethyl benzaldehyde to form the respective tetraphenyl cyclohexenoporphyrins (TPCHPs). These compounds were isolated as dark green cationic salts that were deprotonated to form the corresponding purple freebase species, which were then subsequently metalated with PtCl_2_ and PdCl_2_ to afford the metalated TPCHPs. The metalated TPCHPs were then converted to the benzo derivatives by aromatization with excess DDQ, giving yields of approximately 30% for the Pt(ii) benzoporphyrins and roughly 90% for Pd(ii) benzoporphyrins.

### UV-Vis electronic absorption spectroscopy

**Fig. 2:**
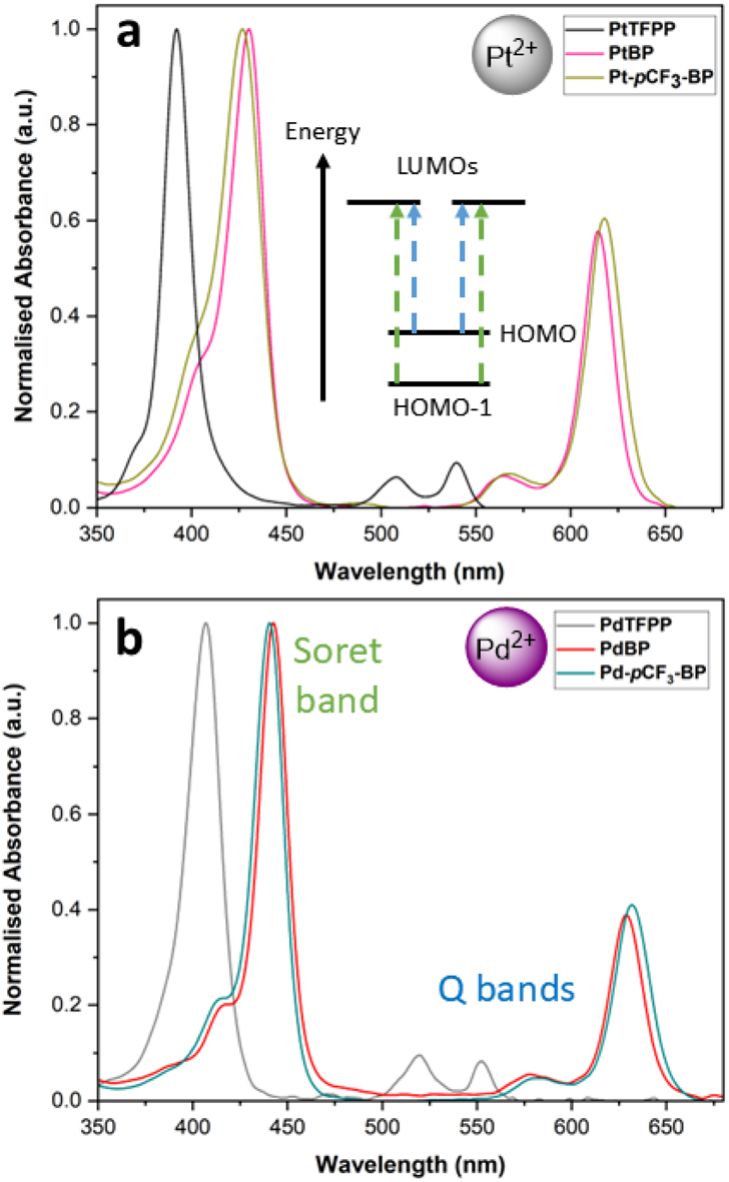
The UV-Vis electronic absorption spectra of (a) PtTFPP,^32^PtBP and Pt-*p*CF_3_-BP and (b) PdTFPP,^33^PdBP and Pd-*p*CF_3_-BP in chloroform. Accompanying this, is a typical energy level diagrams showing the four frontier MOs found in tetraphenyl porphyrins and the electronic transitions that form the Soret and Q band features.

The UV-Vis electronic absorption spectra are characteristic of metalloporphyrins, with an intense feature in the near-UV, called the Soret band and weaker visible region features, called Q bands (Fig. 2) (Table S1†). The higher energy Q band is denoted as *Q*_abs_(1,0) and the lower in energy as *Q*_abs_(0,0).^35^ These spectral features can be assigned according to Gouterman's four-orbital model, with the Soret band being comprised of a pair of symmetry-matched excited states; each being formed predominantly of a one-electron transition from the HOMO−1 to one of the degenerate LUMOs.^36^ Correspondingly, the *Q*_abs_(0,0) feature is formed of a pair of symmetry-matched excited states that are predominantly formed of a one-electron transitions from the HOMO to the one of the degenerate LUMOs. The *Q*_abs_(1,0) feature is a vibronic satellite that is allowed *via* a Herzberg-Teller coupling pathway.^37^ A key distinction of the spectra of the benzoporphyrins compared to the tetraphenyl porphyrins, is the overall red shift of absorption features; as well as the greatly increased intensity of the *Q*_abs_(0,0) feature, which is commonly found throughout the literature.^31^

### Emission spectroscopy

**Fig. 3:**
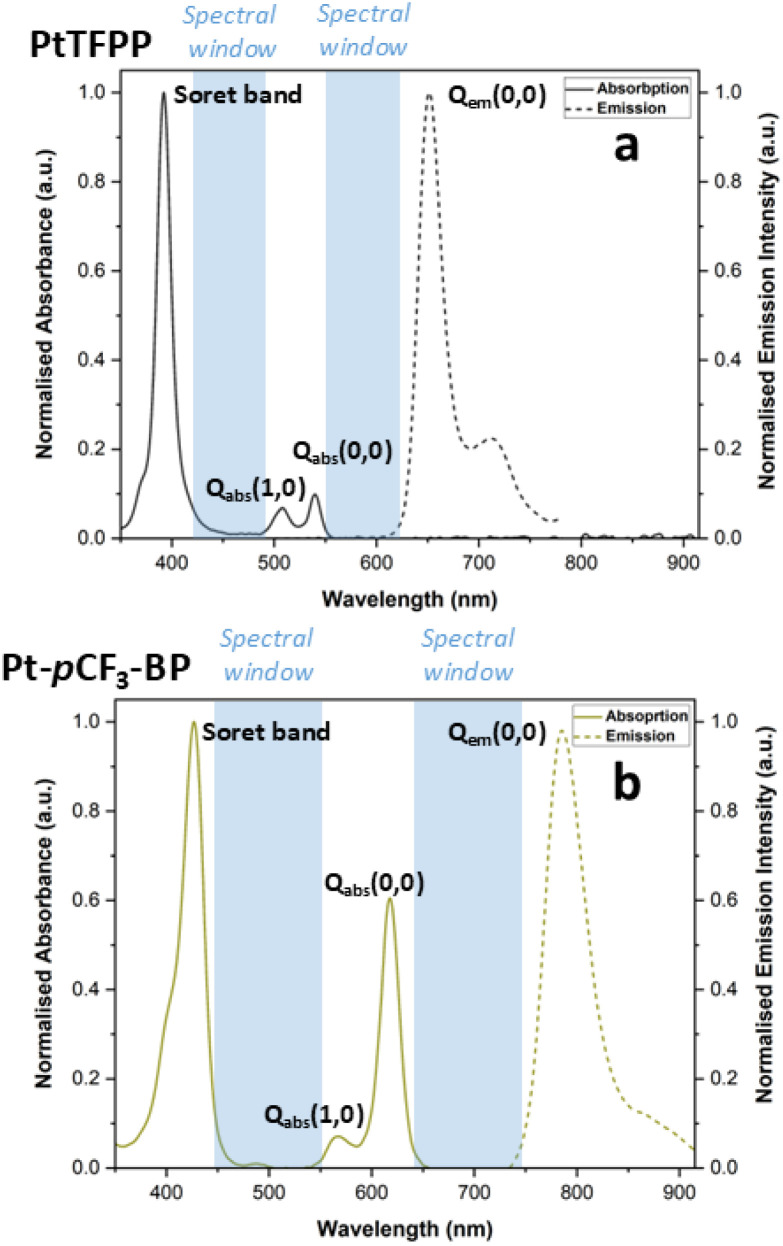
The normalised absorption and emission spectra of (a) PtTFPP and (b) Pt-*p*CF_3_-BP showing the ‘spectral windows’ where a secondary luminophore's emission spectrum could viably occur.

The emission spectra of the porphyrin luminophores possess two distinct characteristic bands, a higher in energy electronic origin band denoted as *Q*_em_(0,0) and a lower in energy vibronic satellite, denoted as *Q*_em_(0,1) (Fig. 3) (Table 1).^35^ The lifetimes of emission, *τ*_(Ar)_ and quantum yields *Φ*_(Ar)_ were measured in argon saturated solutions. Lifetime mono-exponential decay fits and the emission spectra for all the luminophores can be found in the accompanying ESI.

**Table 1:** Emission peak maxima, select spectral window, lifetimes of emission, quantum yields of emission and brightness values in deoxygenated chloroform for the porphyrins

Porphyrin	*Q*_em_(0,0) maximum (nm)	*Q*_em_(0,1) maximum (nm)	Soret band to *Q*_abs_(1,0) spectral window width^c^ (nm)	*Q*_abs_(1,0) to *Q*_em_(0,0) spectral window width^c^ (nm)	Lifetime of emission in argon *τ*_(Ar)_ (μs)	Quantum yield of emission in argon *Φ*_(Ar)_	Brightness BS *Φ*_(Ar)_ × *ε* (M^−1^ cm^−1^)
PtTFPP^a^	651	710	115	111	49.6	0.08	23 700
PtBP	775	—	134	161	48.7	0.55	115 800
Pt-*p*CF_3_-BP	786	—	140	168	47.5	0.63	136 400
PdTFPP^b^	672	739	112	120	748.2	0.04	10 900
PdBP	806	—	135	175	258.7	0.17	44 900
Pd-*p*CF_3_-BP	816	—	142	180	234.3	0.19	52 900

a Data previously published.^32^

b Data previously published.^33^

c Calculated as the difference between the respective features' maxima.

The Pd(ii) benzoporphyrins exhibit the typical spectral red shift (roughly 27 nm) relative to the Pt(ii) analogues. A large red shift (roughly 134 nm) is present for the emission spectra of the benzoporphyrins when compared to their TFPP derivatives. This red shift of the emission features into the NIR region makes the benzoporphyrins viable luminophores for new and improved binary PSP formulations. There are two ideal ‘spectral windows’ in which a secondary temperature-sensitive luminophore could emit to avoid significant spectral overlap with the pressure-sensitive luminophore, the Soret band to the *Q*_abs_(1,0) feature and the *Q*_abs_(0,0) feature to the *Q*_em_(0,0) feature. For the benzoporphyrins, these spectral windows are much wider; for example, for Pt-*p*CF_3_-BP, they are 25 nm and 57 nm wider when compared to PtTFPP. Although this increase in spectral window appears small, using the sharp emission features of lanthanide temperature-sensitive luminophores, this could significantly decrease performance-reducing spectral overlap with the pressure-sensitive luminophore signal. Additionally, the red shifted spectra of the benzoporphyrins recentres the Soret band to *Q*_abs_(1,0) spectral window to roughly 508 nm, putting it in the spectral range of the emission spectrum of the desirable temperature-sensitive luminophore coumarin 6.^19^

Compared to the TFPP complexes, the benzoporphyrins exhibit increases in *Φ*_(Ar)_ (roughly 7× larger for the Pt(ii) and 4× larger for the Pd(ii) benzoporphyrins). Incorporation of the *para* CF_3_ group further increases *Φ*_(Ar)_, with PtBP and Pt-*p*CF_3_-BP possessing *Φ*_(Ar)_ values of 0.55 and 0.63 respectively. This increase in *Φ*_(Ar)_ matches well with Borisov *et al.* who found that *para* fluorination can increase the *Φ*_(Ar)_ of benzoporphyrins, whilst *meta* substitution was found to decrease *Φ*_(Ar)_.^31^PdBP and Pd-*p*CF_3_-BP follow a similar trend. The brightness (BS) of a given luminophore can be determined using the product of the molar absorption coefficient and the quantum yield of emission (BS = *Φ*_(Ar)_ × *ε*). For the benzoporphyrins, the larger quantum yields result in much brighter luminophores when compared to the TFPP complexes; for example, the BS of PtTFPP, PtBP and Pt-*p*CF_3_-BP is 23 700, 115 800 and 136 400 M^−1^ cm^−1^ respectively.

Therefore, the benzoporphyrins are roughly 5× brighter than the TFPP complexes, rendering them attractive new luminophores for PSP formulations.

The lifetimes of emission, *τ*_(Ar)_ for the Pt(ii) benzoporphyrins show a slight decrease from that of PtTFPP; however, the Pd(ii) benzoporphyrins show a much larger reduction in *τ*_(Ar)_ (roughly 2–3× lower) compared to PdTFPP. This large reduction for the Pd(ii) benzoporphyrins, is likely due to their longer lived T_1_ state being more sensitive to the increase in T_1_ → S_0_ non-radiative decay associated with the large out-of-plane structural distortions adopted by the benzoporphyrins.^38^

### Polystyrene PSP performance studies

**Fig. 4:**
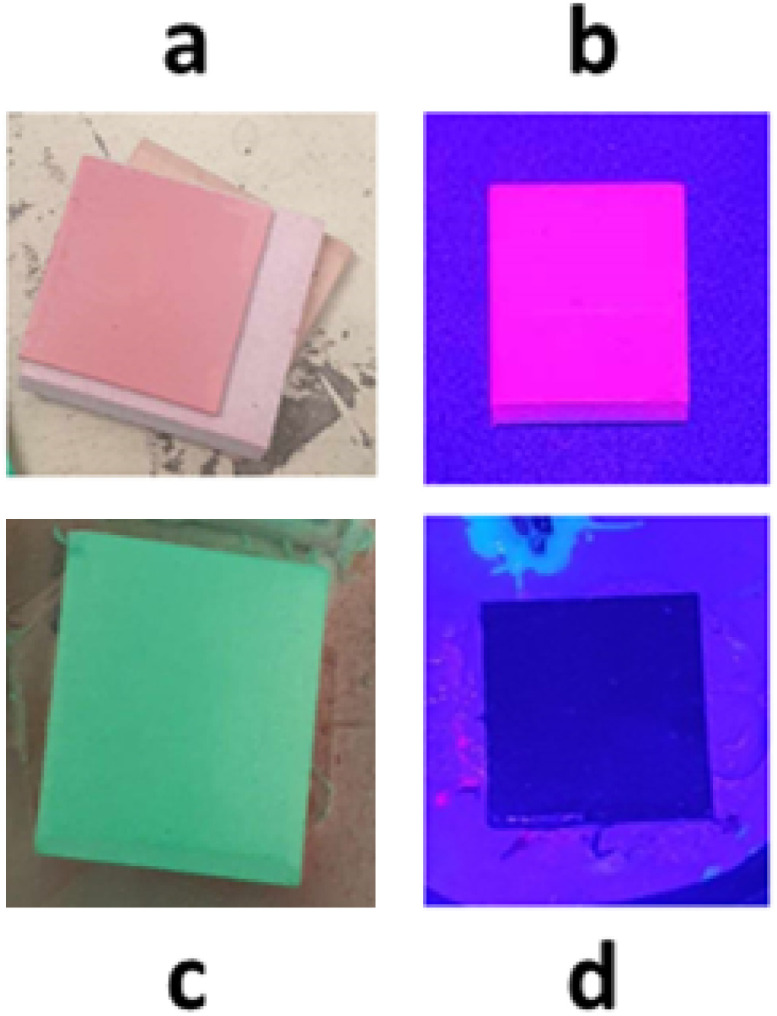
Images of select PSP samples. (a) Traditional PtTFPP-based PSP. (b) PtTFPP-based PSP under light excitation. (c) Benzoporphyrin based PSP. (d) Benzoporphyrin based PSP under light excitation.

The benzoporphyrins and Pt/PdTFPP were trialled in simple polystyrene PSPs, using an *a priori* calibration method described in our previous work.^32^ The polystyrene formulation was used in our previous studies and is effective for direct luminophore comparison (see ESI† for full details).^32^ These samples are denoted as luminophore (PS). One noteworthy feature of the benzoporphyrin-based PSPs is their unique green colour, in contrast to the pink colour of Pt/PdTFPP based PSPs, along with their black appearance under 430 nm light excitation (Fig. 4).

A crucial performance metric for PSPs is the pressure sensitivity (*S*_p_). A large *S*_p_ allows smaller pressure changes to be more accurately resolved; however, too high a pressure sensitivity can result in excessive quenching and therefore a loss of performance at higher pressures. *S*_p_ is governed by two factors, the oxygen permeability of the binder matrix, and the lifetime of the luminophore excited state, *τ*_(Ar)_ (from the Stern–Volmer constant (*K*_SV_ = *k*_q_*τ*_0_). If the binder is kept the same the effect of the luminophore species on *S*_p_ can be isolated. In general, a longer-lived luminophore excited state has a higher chance of undergoing collisional quenching with oxygen and therefore, affords an increased *S*_p_.

**Fig. 5:**
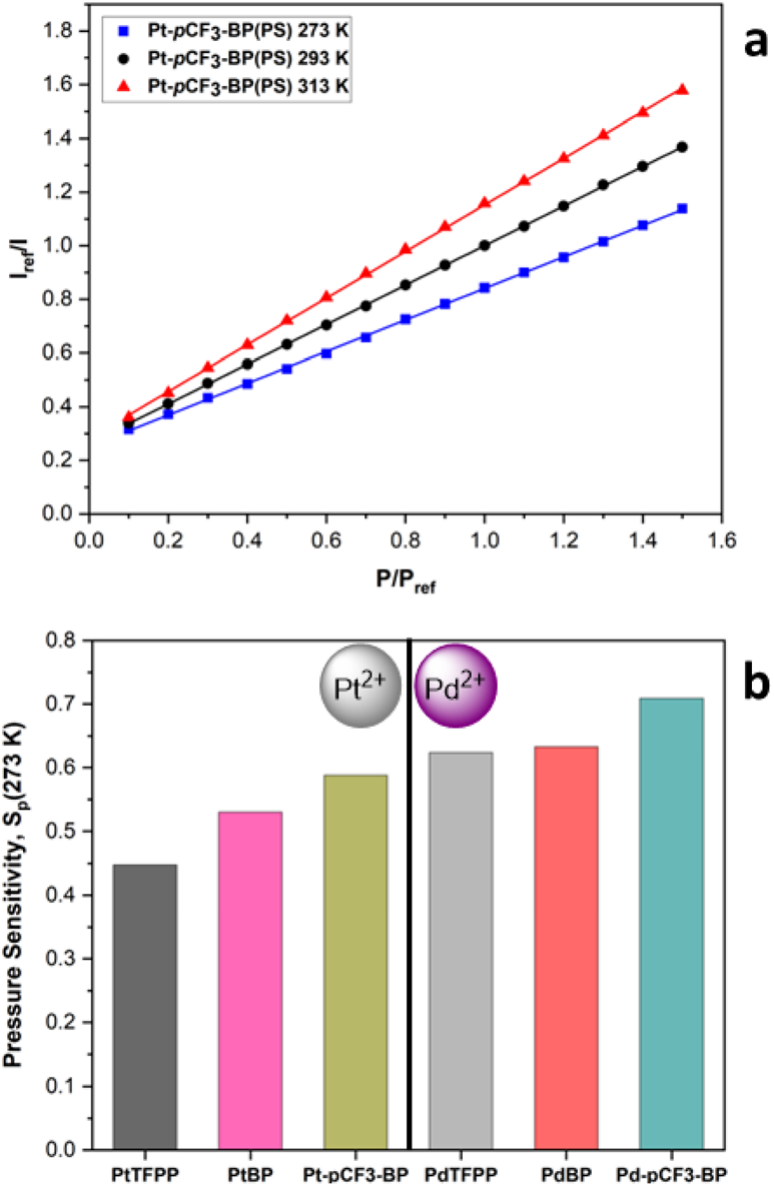
(a) The modified Stern–Volmer calibrated luminescence response to pressure with associated linear first for Pt-*p*CF_3_-BP(PS) at 273 K (*R*^2^ = 1), 293 K (*R*^2^ = 1) and 313 K (*R*^2^ = 1). (b) The pressure sensitivity at 273 K, *S*_p_ (273 K), for the porphyrin polystyrene PSPs. The *S*_p_ is calculated using the linear form of the modified Stern–Volmer (eqn S(1)†). The data for PtTFPP and PdTFPP is previously published.^33^

For all the polystyrene PSPs, a linear modified Stern–Volmer fit (eqn S(1)†) (Fig. 5a) was used to determine *S*_p_; however, some non-linearity is present in the Pd(ii) porphyrin polystyrene-based PSPs due their longer phosphorescence lifetimes.

The individual modified Stern–Volmer calibrated plots for each polystyrene PSP at 273, 293 and 313 K and the *S*_p_ at each temperature can be found in the accompanying ESI. The Pt(ii) benzoporphyrin polystyrene-based PSPs exhibit remarkable linearity in their Stern–Volmer response (*R*^2^ = 1), even at higher temperatures where non-linear behaviour is typical for PtTFPP. Focusing on the Pt(ii) porphyrins first, the *S*_p_ at 273 K denoted as *S*_p_(273 K) (Fig. 5b) increases from 0.448 for PtTFPP(PS)^33^ to 0.530 for PtBP(PS) and then to 0.588 for Pt-*p*CF_3_-BP(PS). The same trend occurs for the Pd(ii) analogues with *S*_p_(273 K) increasing from 0.624 for PdTFPP(PS) to 0.633 for PdBP(PS) and then to 0.709 for Pd-*p*CF_3_-BP(PS); however, the Pd(ii) porphyrins exhibited a larger *S*_p_, owing to their significantly longer *τ*_(Ar)_.

Overall, the benzoporphyrin polystyrene-based PSPs are more sensitive to pressure than Pt/PdTFPP(PS). The highest *S*_p_(293 K) is found for the *para* CF_3_ substituted benzoporphyrins, Pt/Pd-*p*CF_3_-BP(PS), agreeing with our previous study on tetraphenyl porphyrins, which suggested that CF_3_ groups on the luminophore can increase *S*_p_ in polymer-based PSPs.^32^ The relative emission lifetimes of the luminophores are similar in polystyrene and solution (Table S2†) – implying a large increase in emission lifetime does not correlate with the much higher pressure sensitivities of the benzoporphyrin-based polystyrene PSPs. At 293 K and 313 K the trends in *S*_p_ break down as the individual temperature sensitivities of each luminophore start to increasingly influence the *S*_p_ of the PSPs. The increased brightness of emission, wider spectral windows and higher-pressure sensitivities of the benzoporphyrin polystyrene-based PSPs, make them attractive options as new luminophores in PSP formulations.

**Fig. 6:**
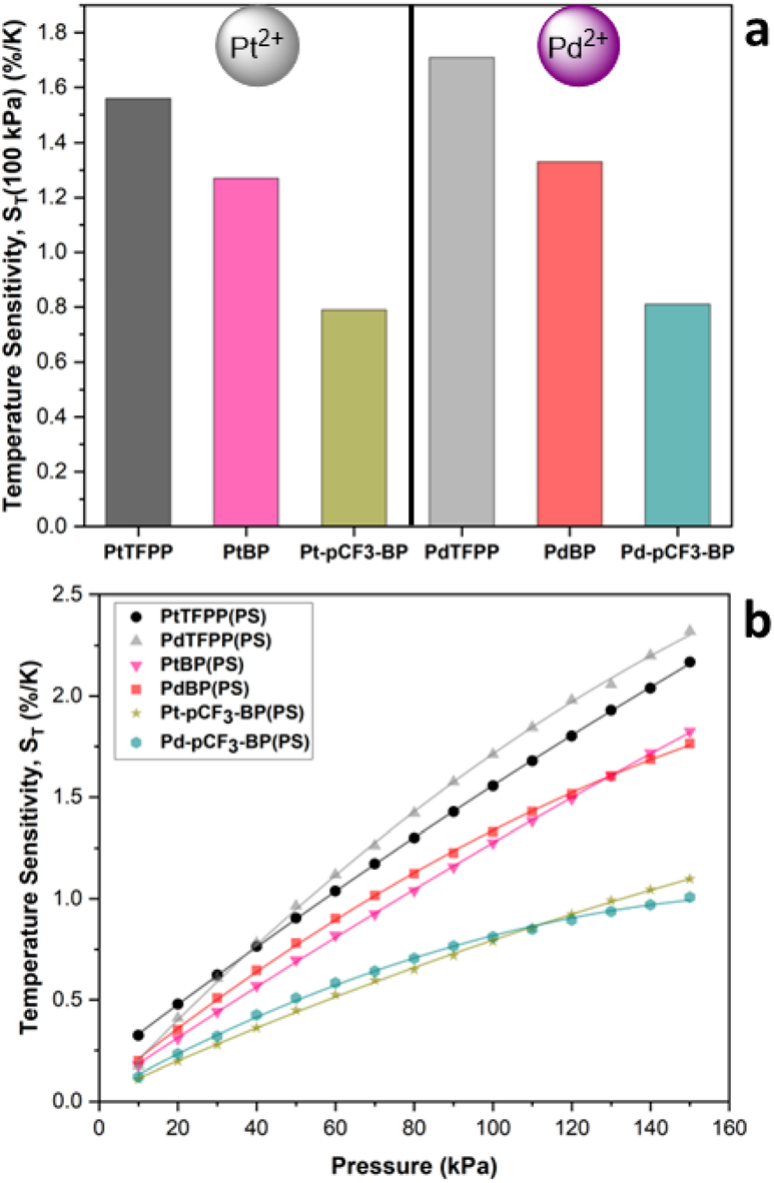
(a) The *S*_T_ at 100 kPa, *S*_T_(100 kPa) for the polystyrene-based PSPs. (b) The change in *S*_T_ with pressure for PtTFPP, PdTFPP, PtBP, PdBP, Pt-*p*CF_3_-BP and Pd-*p*CF_3_-BP polystyrene-based PSPs. The data for PtTFPP and PdTFPP is previously published.^33^

PSPs also have an inherent temperature sensitivity (*S*_T_), experiencing a reduction in luminescence intensity with increasing temperature. Ideally, a PSP formulation should have zero temperature dependence. A large S_T_ can make accurate determination of pressure challenging or even impossible when surface temperature gradients are also present. Recently, we demonstrated that the extent, pattern and nature of phenyl halogenation for tetraphenyl porphyrin luminophores can have a significant effect on the *S*_T_ of polymer-based PSPs.^32^ Consequently, we then investigated the effect of the nature of the central metal ion for TFPP luminophores on polymer-based PSP performance, finding that PtTFPP(PS) had a lower *S*_T_ at higher pressures and PdTFPP(PS) had a lower *S*_T_ at lower pressures.^33^ The *S*_T_ for a given PSP is calculated using eqn (S3).† Focusing on the *S*_T_ at 100 kPa (Fig. 6a), denoted as *S*_T_(100 kPa); for PtBP(PS) (1.27%/K) and Pt-*p*CF_3_-BP(PS) (0.79%/K), they both possess a lower *S*_T_(100 kPa) than that of PtTFPP(PS) (1.56%/K).^33^ The same trend occurs for the Pd(ii) benzoporphyrin polystyrene-based PSPs, but *S*_T_ is generally higher when compared to the Pt(ii) analogues. For example, for PdBP(PS) (1.33%/K) and Pd-*p*CF_3_-BP(PS) (0.81%/K), they both have a lower *S*_T_(100 kPa) than that of PdTFPP(PS) (1.71%/K).^33^ Overall, the unsubstituted benzoporphyrins reduce *S*_T_ by 20% and the *para* CF_3_ substituted benzoporphyrins by 50% from that of the Pt/PdTFPP(PS). This significantly decreased *S*_T_ makes the *para* CF_3_ substituted benzoporphyrins excellent candidates for improved PSPs.

The change in *S*_T_ with pressure, for a given PSP, is also important to consider (Fig. 6b). *S*_T_ increases with increasing pressure because of the increasing concentration of the quencher oxygen molecules. An ideal PSP would have a low *S*_T_ that remains constant with pressure. *S*_T_ is observed to change most drastically (steeper gradient) with pressure for PtTFPP(PS) and PdTFPP(PS).^33^ For the *para* CF_3_ substituted benzoporphyrins, *S*_T_ increases at a slower rate (shallower gradient) with increasing pressure than the unsubstituted benzoporphyrins and the TFPP derivatives, resulting in better sensor performance. The reason for the lower sensitivity of *S*_T_ to pressure may be due to increased luminophore compatibility with the polystyrene matrix for Pt/Pd-*p*CF_3_-BP, because of the increased solubility afforded by the CF_3_ substituents. The increased pressure sensitivity of the Pt/Pd-*p*CF_3_-BP PSPs, compared to the Pt/PdBP PSPs, supports this conclusion. For the Pd(ii) luminophores, *S*_T_ is more sensitive to increasing pressure, with a highly non-linear response, when compared to their Pt(ii) analogues. The highly non-linear behaviour of *S*_T_ for the Pd(ii) analogues, is likely due to the longer lived excited state of the Pd(ii) benzoporphyrins being more sensitive to increasing pressure. Eventually, at higher pressures, *S*_T_ for the Pd(ii) benzoporphyrin PSPs begins to plateau (possibly due to excess quenching) and drop below that of their corresponding Pt(ii) luminophores. This plateauing occurs at increasingly higher pressures, for example: Pd-*p*CF_3_-BP(PS) at 110 kPa, PdBP(PS) at 130 kPa and then at >150 kPa for PdTFPP(PS) – aligning well with the increasing luminescence lifetime across this series. The initial rise in *S*_T_ at lower pressures is greatest for PdTFPP(PS) and is again most likely due to the longer *τ*_(Ar)_ of PdTFPP > PdBP > Pd-*p*CF_3_-BP.

### FIB PSP performance studies

To explore the efficacy of benzoporphyrin luminophores in PSP further, Pt-*p*CF_3_-BP and Pd-*p*CF_3_-BP were made into fluoro/iso/butyl polymer (FIB polymer) formulations denoted as Pt-*p*CF_3_-BP(amount of dye FIB) and Pd-*p*CF_3_-BP(amount of dye FIB). FIB polymer was first introduced by Puklin *et al.* as a low temperature sensitivity polymer for PSP, due to its low energy barrier to oxygen diffusion.^39^ The CF_3_ substituted benzoporphyrins were chosen as they are higher performing than the unsubstituted benzoporphyrins. Additionally, it was anticipated that the CF_3_ groups would induce more compatibility between the highly polar FIB polymer binder and the luminophore. The *S*_p_ at each temperature and the *S*_T_(100 kPa) for each FIB PSP can be found in the accompanying ESI (Tables S3 and S4†).

**Fig. 7:**
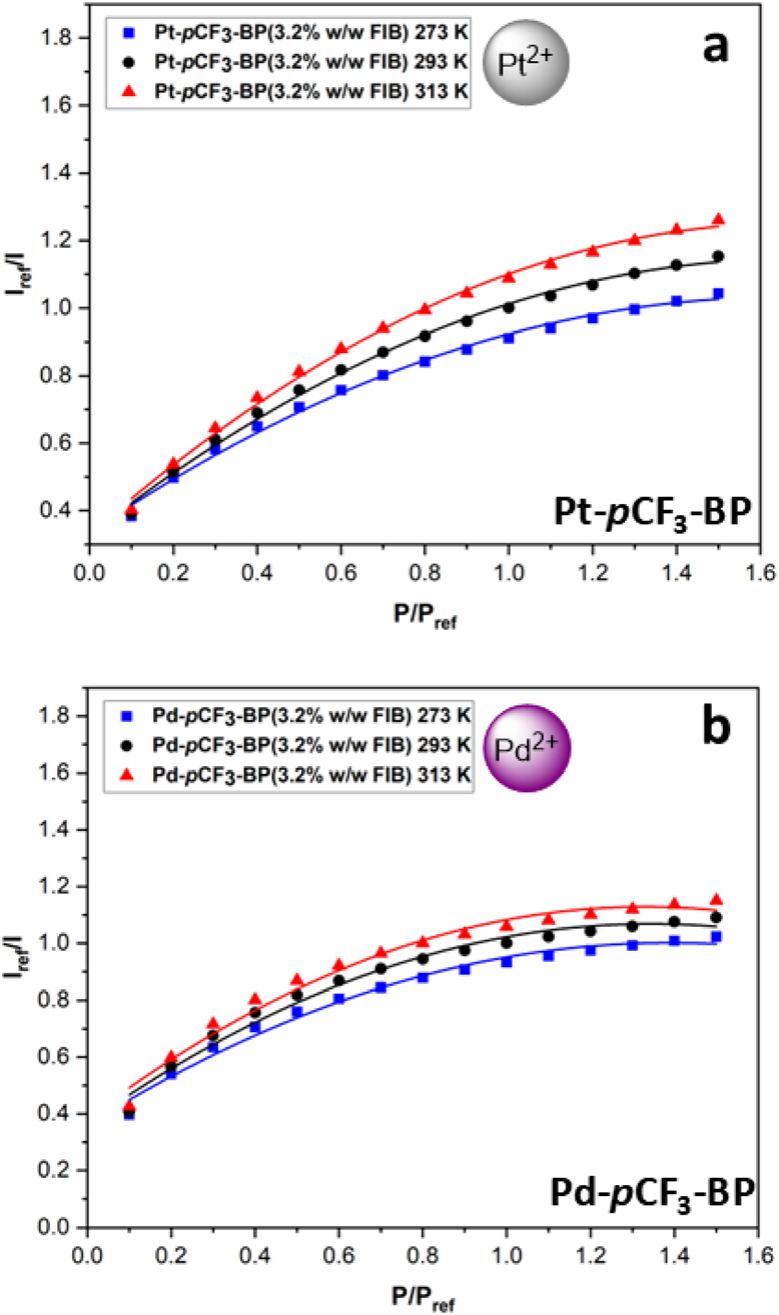
The modified Stern–Volmer calibrated luminescent response to pressure with 2nd order polynomial fits (eqn S(2)†) at 273 K, 293 K and 313 K for (a) Pt-*p*CF_3_-BP(3.2% w/w FIB) (average *R*^2^ = 0.995). (b) Pd-*p*CF_3_-BP(3.2% w/w FIB) (average *R*^2^ = 0.980). The average *R*^2^ is across the fits for the three temperatures.

Initially an in-house FIB recipe was used as a trial for FIB-based benzoporphyrin PSP that was optimised for tetra-aryl metalloporphyrins in our previous work. This involved using 3.2% w/w dye to FIB polymer and 3.2% w/v FIB polymer to trifluorotoluene (TFT).^32^ A smooth coating was acquired after 10 light coats of spray brushing, which was subsequently cured in an oven for two hours to reach the *T*_g_ of FIB at 75 °C. However, when the samples were placed into the calibration chamber and illuminated under 430 nm light, no signal could be detected. After increasing the exposure time of the camera to 400 000 μs for Pt-*p*CF_3_-BP(3.2% w/w FIB) and 800 000 μs for Pd-*p*CF_3_-BP(3.2% w/w FIB), a weak signal was observed. For comparison, the benzoporphyrin polystyrene PSPs displayed an excellent signal at 4000 μs exposure time on the same camera. The performance of the PSPs (Fig. 7) was similarly poor with low pressure sensitivities (especially at higher pressures) that are highly non-linear compared to the polystyrene PSPs (the non-linear Stern–Volmer eqn (S2)† better fits the data).

The poor performance of Pt/Pd-*p*CF_3_-BP in FIB could be due to several reasons. The low luminescence signal implies too high a concentration of luminophore was used and therefore, significant self-quenching is occurring (sensor deactivation). Grenoble *et al.* observed an increased susceptibility of PtOEP to undergo self-quenching over PtTFPP.^26^ As previously mentioned, we used an in-house recipe developed for platinum(ii)-5,10,15,20-tetrakis-(3,5-bis(trifluoromethyl)phenyl)-porphyrin and therefore, the benzoporphyrins appear to be more sensitive to concentration quenching. Additionally, the highly non-linear response of Pt/Pd-*p*CF_3_-BP(3.2% w/w FIB) to pressure, implies a low level of luminophore-polymer compatibility. Therefore, this low level of luminophore-polymer compatibility, results in the Pt/Pd-*p*CF_3_-BP being potentially unsuitable for FIB-based PSP formulations

**Fig. 8:**
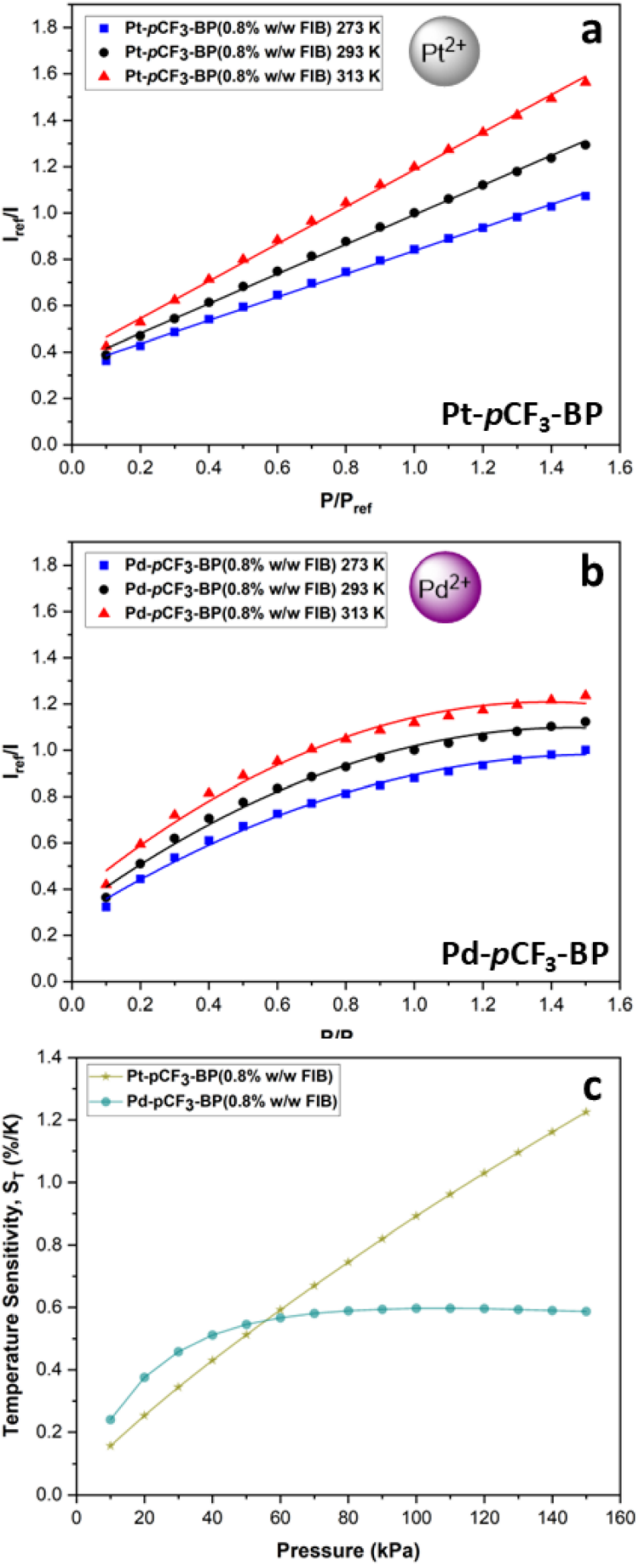
The modified Stern–Volmer calibrated luminescent response to pressure at 273, 293 and 313 K for (a) Pt-*p*CF_3_-BP(0.8% w/w FIB) with linear fits (eqn S(1)†) (average *R*^2^ = 0.998). (b) Pd-*p*CF_3_-BP(0.8% w/w FIB) with 2nd order polynomial fits (eqn S(2)†) (average *R*^2^ = 0.990). (c) The change in *S*_T_ with increasing pressure for Pt-*p*CF_3_-BP(0.8% w/w FIB) and Pd-*p*CF_3_-BP(0.8% w/w FIB). The average *R*^2^ is across the fits for the three temperatures.

To eliminate the observed self-quenching in benzoporphyrin FIB-based PSPs, a lower luminophore loading was examined. A formulation of 0.8% w/w luminophore to FIB polymer and 3.2% w/v FIB polymer to TFT was used. These samples were sprayed in 10 light coats and cured in an oven for two hours to reach the *T*_g_ of FIB at 75 °C. The samples are denoted as Pt-*p*CF_3_-BP(0.8% w/w FIB)/Pd-*p*CF_3_-BP(0.8% w/w FIB). An intense signal with a 10 000 μs camera exposure time could be achieved for both Pt-*p*CF_3_-BP(0.8% w/w FIB) and Pd-*p*CF_3_-BP(0.8% w/w FIB). This significant increase in brightness compared to the higher luminophore loading samples, suggests that self-quenching had effectively been reduced by using the lower luminophore loading. The performance of the benzoporphyrin FIB-based PSPs, using the lower luminophore loading, was also significantly improved (Fig. 8). For example, the *S*_p_(293 K) of Pt-*p*CF_3_-BP(0.8% w/w FIB) is 0.501 and the response to pressure was much more linear (*R*^2^ = 0.998 using the linear Stern–Volmer eqn S(1)†) when compared to Pt-*p*CF_3_-BP(3.2% w/w FIB). However, the pressure response of Pt-*p*CF_3_-BP(0.8% w/w FIB) is still less linear than Pt-*p*CF_3_-BP(PS). The *S*_T_(100 kPa) of Pt-*p*CF_3_-BP(0.8% w/w FIB) compared to Pt-*p*CF_3_-BP(PS) is higher (0.89%/K and 0.79%/K respectively). The FIB polymer has a low energy barrier to oxygen diffusion and thus is expected to reduce the *S*_T_ of a given PSP.^39^ Additionally, the *S*_T_ of Pt-*p*CF_3_-BP(0.8% w/w FIB) is much more sensitive to pressure (Fig. 8c) when compared to Pt-*p*CF_3_-BP(PS). Overall, these factors result in Pt-*p*CF_3_-BP(0.8% w/w FIB) exhibiting reduced performance compared to Pt-*p*CF_3_-BP(PS), which suggests a level of incompatibility between the partially fluorinated Pt-*p*CF_3_-BP and the highly polar FIB polymer. For example, the heavily fluorinated platinum(ii)-5,10,15,20-tetrakis-(3,5-bis(trifluoromethyl)phenyl)-porphyrin luminophore we recently trialled in FIB, displayed excellent linearity in its response to pressure and much higher performance metrics.^32^ The performance of Pd-*p*CF_3_-BP(0.8% w/w FIB) was still poor, compared to Pd-*p*CF_3_-BP(3.2% w/w FIB), with a highly non-linear pressure response and low pressure sensitivities. The long *τ*_(Ar)_ of Pd-*p*CF_3_-BP is probably too sensitive for the FIB binder, resulting in an over-sensitivity to pressure. The low activation energy of oxygen diffusion for the FIB polymer results in higher concentrations of oxygen dissolved in the polymer, which will affect Pd-*p*CF_3_-BP more than Pt-*p*CF_3_-BP due to the much longer *τ*_(Ar)_ of the former.

## Conclusions

Benzoporphyrins are an attractive class of luminophore for a new and improved generation of NIR-emitting polymer-based PSP formulations, which exhibit more desirable properties over PSPs utilising Pt/PdTFPP. In particular, the wider and red shifted spectral windows in the benzoporphyrin's absorption and emission spectra allow more spectral room for a secondary temperature sensitive luminophore, thus potentially decreasing performance reducing spectral overlap and crosstalk in future binary PSPs. The benzoporphyrin luminophores offer increased luminescent brightness; in particular, the *para* CF_3_ substituted benzoporphyrins exhibited a roughly 5× higher brightness compared to the traditional Pt/PdTFPP luminophores. Initial polystyrene PSP trials, using *para* CF_3_ substituted benzoporphyrins, afforded PSPs with significantly higher performance metrics. For example, the benzoporphyrins increased pressure sensitivity, *S*_p_, by 20% and reduced temperature sensitivity, *S*_T_, by 50% when compared to Pt/PdTFPP polystyrene-based PSPs. The *para* CF_3_ substituted benzoporphyrins were examined in the higher performing fluoro/Iso/butyl (FIB) polymer. The benzoporphyrins were found to be concentration sensitive which reduced brightness and PSP performance at higher luminophore loadings. Using a lower luminophore loading, the benzoporphyrin FIB-based PSPs exhibited a reasonable performance; however, some performance loss was observed when compared to the polystyrene PSPs. This performance loss is theorized to be caused by a lower luminophore-polymer compatibility between the partially fluorinated benzoporphyrin and the highly fluorinated FIB polymer.

In general, the benzoporphyrins present a significant performance improvement over the traditional Pt/PdTFPP luminophores in polystyrene PSPs and thus are attractive candidates for new brighter NIR-emitting PSP formulations. We plan to test the benzoporphyrins presented here, in a range of PSP formulations, including binary systems, to fully explore their efficacy as new NIR-emitting luminophores for improved PSP formulations in wind tunnel tests. We hope this investigation inspires further development of NIR-emitting luminophores as they offer distinct advantages over the widely used traditional luminophores, particularly in the development of higher performing binary PSPs.

## Data availability

The authors confirm that the data supporting the findings of this study are available within the article and/or its ESI.

## Author contributions

Elliott J Nunn: writing – review and editing, writing – original draft, visualization, methodology, data curation, conceptualization. Dimitrios Tsioumanis: visualization, methodology, data curation. Tom B. Fisher: writing – review and editing, supervision, funding acquisition, conceptualization. David. A. Roberts: writing – review and editing, supervision, funding acquisition, conceptualization. Mark K. Quinn: writing – review and editing, validation, supervision, project administration, funding acquisition, conceptualization. Louise S. Natrajan: writing – review and editing, validation, supervision, project administration, funding acquisition.

## Conflicts of interest

There are no conflicts of interest to declare

## Supplementary Material

SC-016-D5SC00810G-s001
