# Supplementary material for: Development of bright NIR-emitting pressure-sensitive paints using benzoporphyrin luminophores
Source: Chem Sci. 2025 Mar 20;16(16):7018–25. doi: 10.1039/d5sc00810g (PMC11933923; doi:10.1039/d5sc00810g)
Supplement: SC-016-D5SC00810G-s001 [file SC-016-D5SC00810G-s001.pdf]

Supplementary Information (SI) for Chemical Science.
This journal is © The Royal Society of Chemistry 2025

Supporting Information

Development of bright NIR emitting pressure-sensitive paints using benzoporphyrin luminophores

Table of Contents

1. Photophysical studies 2
2. PSP recipes 2
3. PSP performance studies 3
4. Synthesis 4
4.1 General synthetic details 4
4.2 Synthetic route 5
4.3 Synthetic procedures 6
4.3.1 Synthesis of 2 6
4.3.2 Synthesis of 3 6
4.3.3 Synthesis of 4a and 4b 7
4.3.4 Synthesis of Pt4a, Pd4a, Pt4b and Pd4b 8
4.3.5 Synthesis of PtBP, PdBP, Pt-*p*CF_3-BP and Pd-*p*CF_3-BP 10
4.4 Characterisation data 12
4.4.1 Characterisation data of 2 12
4.4.2 Characterisation data of 4a 12
4.4.3 Characterisation data of 4b 14
4.4.4 Characterisation data of Pt4a 16
4.4.5 Characterisation data of Pd4a 18
4.4.6 Characterisation data of Pt4b 20
4.4.7 Characterisation data of Pd4b 23
4.4.8 Characterisation data of PtBP 26
4.4.9 Characterisation data of PdBP 28
4.4.10 Characterisation data of Pt-*p*CF_3-BP 29
4.4.11 Characterisation data of Pd-*p*CF_3-BP 32
5. UV-Vis electronic absorption spectroscopy 35
6. Lifetimes of Emission 35
7. Emission Spectra 36
8. Polystyrene PSP performance studies 37
9. FIB PSP performance studies 39
10. References 40

1. Photophysical studies

UV-vis electronic absorption spectra were recorded on a Mettler Toledo UV5Bio spectrophotometer. Steady-state emission and excitation spectra and luminescent lifetime data were recorded on an Edinburgh Instruments FP920 Phosphorescence Lifetime Spectrometer equipped with a 450 W steady-state xenon lamp, a 5 W microsecond pulsed xenon flash lamp, (with single 300 mm focal length excitation and emission monochromators in Czerny-Turner configuration), and interchangeable EPL pulsed diode lasers, and a red-sensitive photomultiplier in Peltier (air-cooled) 53 housing (Hamamatsu R928P). Plotting, fitting, and analysis of data was carried out using Origin 2022b. All data were fitted with exponential decay models and the goodness of fit evaluated by the residual, χ^2 and R^2 analysis. These measurements were recorded in chloroform which was dried over 4 Å molecular sieves and degassed using three freeze pump thaw cycles and standard Schlenk techniques. All samples were prepared in an Innovative Technologies System Two glovebox under argon where the concentration of oxygen and water was always kept below 0.1 ppm. Quantum yields were calculated using the relative method, with tetraphenyl porphyrin in toluene (Φ = 0.07) as a standard.[1] The polystyrene-doped samples were prepared by drop casting approximately 100 µl of the PSP polystyrene solution in chloroform onto a glass microscope slide and air dried. These samples were subsequently taken into an argon-filled glove box to remove all residual volatiles and sealed with another glass slide using vacuum grease around the edges to prevent diffusion of oxygen into the sample. The reported data are an average of three independent measurements.

2. PSP recipes

The polystyrene-based PSPs were formulated with a recipe of 0.64% w/w luminophore to polystyrene and 4% w/v polystyrene) to chloroform. The polystyrene was purchased from Sigma Aldrich (M_w ~ 380,000 kDa)

The FIB based PSPs were formulated into a recipe with 0.8% or 3.2% w/w luminophore to FIB whilst keeping other components consistent, with: 0.48% w/w TiO_2 to FIB and 3.2% w/v FIB to trifluorotoluene.

Detailed information about the FIB polymer used in this study were reported previously.[2]

3. PSP performance studies

PSP formulations were sprayed onto ambersil matt white RAL 9010 base coated aluminium coupons using a spray gun in 10 light coats. Freshly sprayed samples were left to air dry for 30 minutes. The average thickness of the PSP samples was 18 µm, which was determined using an ATP ADT-156 data logging coating thickness meter.

The performance of the PSP formulations was investigated in the standard approach of *a-priori* calibration using the University of Manchester PSP calibration chamber. Detailed procedures for the *a-priori* calibration is previously published.[2] For **PtTFPP** and **PdTFPP** PSPs, illumination was provided by an ISSI air-cooled 5 W UV LED lamp which was left to settle to thermal equilibrium for five minutes before image acquisition (with the sample covered to avoid photodegradation). For any benzoporphyrin-based PSP, illumination was provided by an air-cooled Lumixtar 30 W 430 nm high power LED.[3]

The pressure sensitivity at a certain temperature $S_P(T)$ was calculated from the slope of the modified Stern-Volmer calibration plots using Equation S1. With I_{ref} and P_{ref} being the luminescent intensity and pressure at 100 kPa and 293 K.

$$\frac{I_{ref}}{I} = S_P(T)\frac{P}{P_{ref}} + C(T)$$

(S1)

For some of the PSPs a 2nd order polynomial, Equation S2, was employed to better fit the data.

$$\frac{I_{ref}}{I} = A(T)\left(\frac{P}{P_{ref}}\right)^2 + B(T)\frac{P}{P_{ref}} + C(T)$$

(S2)

The temperature sensitivity at a given pressure $S_T(P)$ is calculated as the percentage change in I_{ref}/I with respect to the temperature using Equation S3. With I_{ref} being the luminescent intensity at 100 kPa and 293 K.

$$\frac{I_{ref}}{I} = S_T(P)T + C(P)$$

(S3)

4. Synthesis

4.1 General synthetic details

All reagents were used as purchased. All NMR Spectra were obtained using either a Bruker Avance III 500 MHz Prodigy instrument or a Bruker Avance III 400 MHz Prodigy instrument at the University of Manchester. Chemical shifts are recorded in parts per million (ppm) from high to low frequency and referenced to the residual solvent resonance. Coupling constants (J) are reported in Hertz (Hz) and splitting patterns are designated as follows: b = broad, s = singlet, d = doublet, t = triplet, q = quartet, p = pentet and m = multiplet. Some specific signals were discerned from the HMBC spectra and are designated by a HMBC in their NMR data. Mass Spectra were obtained through the Mass Spectrometry services in the department of chemistry at the University of Manchester. Column chromatography was performed using Silica gel (sigma-aldrich high-purity grade, pore size 60 Å, 230-400 mesh particle size, 40-63 µm particle size, for flash chromatography)

The characterisation data for **PtTFPP**[2] and **PdTFPP**[4] has been reported previously.

4.2 Synthetic route

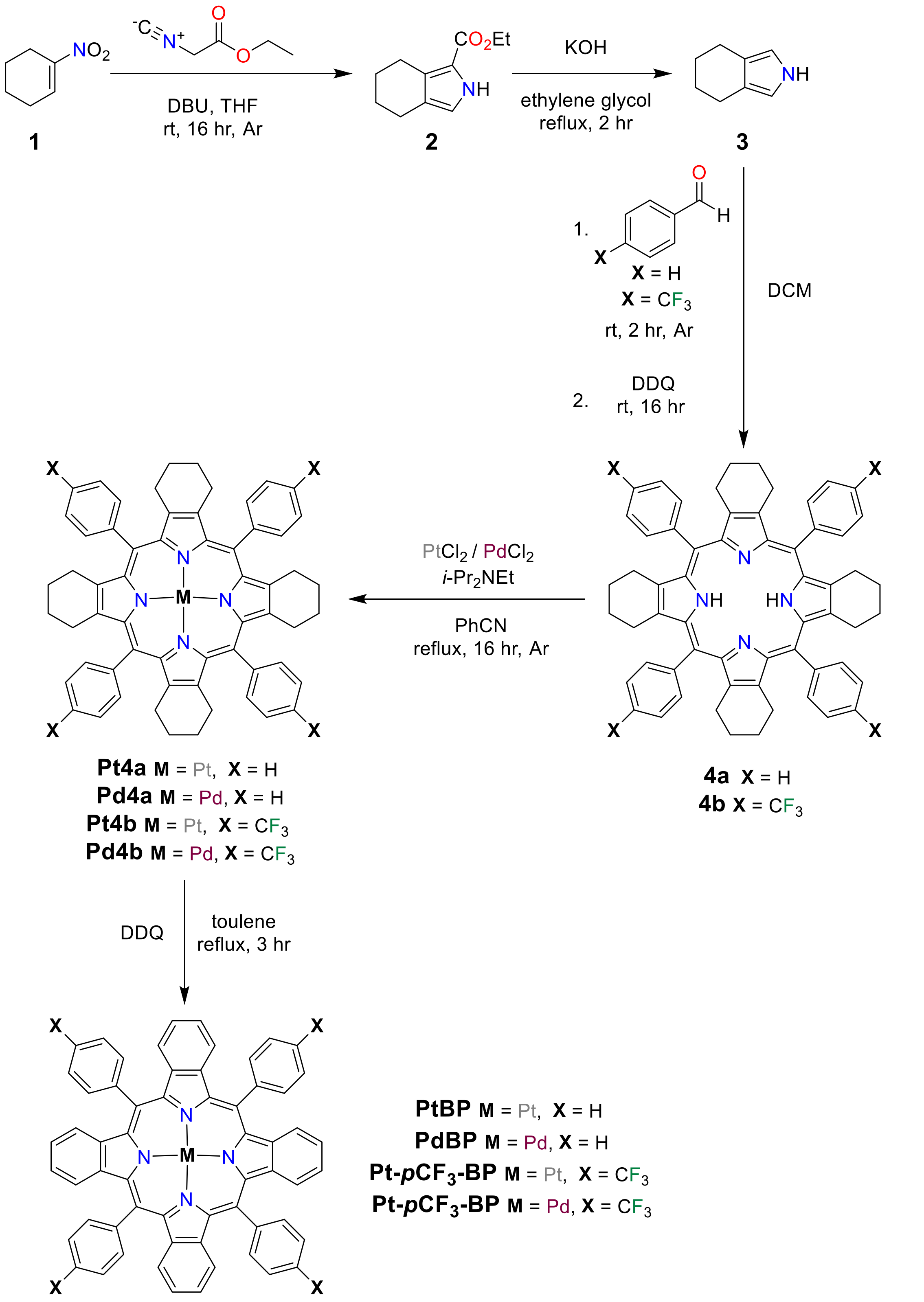

Scheme S1. The synthetic route for the benzoporphyrin synthesis. This synthetic route was first reported by Finikova *et al*.[5]

4.3 Synthetic procedures

4.3.1 Synthesis of 2

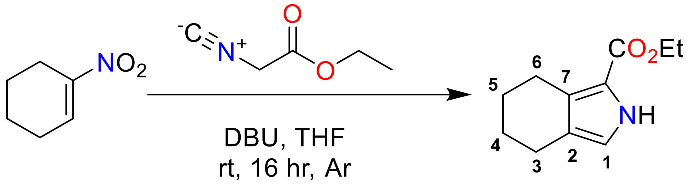

1 **2**

1-Nitrocyclohexene, **1** (4.43 mL, 39.3 mmol) and ethyl isocyanoacetate (4.29 mL, 39.3 mmol) were dissolved in THF (80 mL) and bubbled through with argon for 30 minutes. DBU (5.87 mL, 39.3 mmol) was then added and the reaction stirred at room temperature under argon for 16 hours. The solvent was then removed *via* rotary evaporation and the crude product purified by column chromatography (100% Et_2O) to afford pale yellow crystals, **2** (6.54 g, 31.7 mmol, 81%).

^{1}H NMR (400 MHz, $CDCl_3$) δ = 8.73 (br, 1H, N**H**), δ = 6.64 (d, $^{3}J_{H\text{-}H}$ = 3.2 Hz, 1H, α-pyrrole-1-**H**), δ = 4.29 (q, $^{3}J_{H\text{-}H}$ = 7.1 Hz, 2H, O-C**H₂**-CH₃), δ = 2.81 (t, $^{3}J_{H\text{-}H}$ = 6.0 Hz, 2H, 6-**H**), δ = 2.54 (t, $^{3}J_{H\text{-}H}$ = 6.0 Hz, 2H, 3-**H**), δ = 1.80-1.66 (m, 4H, 4 and 5-**H**), δ = 1.34 (t, $^{3}J_{H\text{-}H}$ = 7.2 Hz, 3H, O-CH₂-C**H₃**).

4.3.2 Synthesis of 3

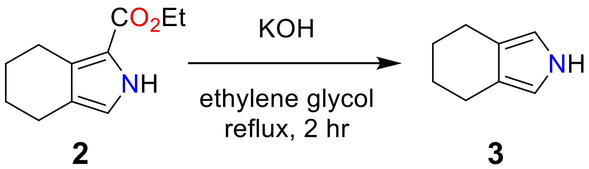

2 **3**

2 (4.50 g, 21.8 mmol) and KOH (18.80 g, 334.2 mmol) were added to ethylene glycol (180 mL) and heated at reflux for two hours. The resulting black solution was immediately cooled in an ice bath. DCM (100 mL) was added and the organic phase washed with water (3 x 75 mL). The combined organic phase was then washed with brine (75 mL) and dried over Na_2SO_4. The solvent was then removed by rotary evaporation affording a black solid (2.90 g). This black solid was used in the next step immediately without any further purification.

4.3.3 Synthesis of 4a and 4b

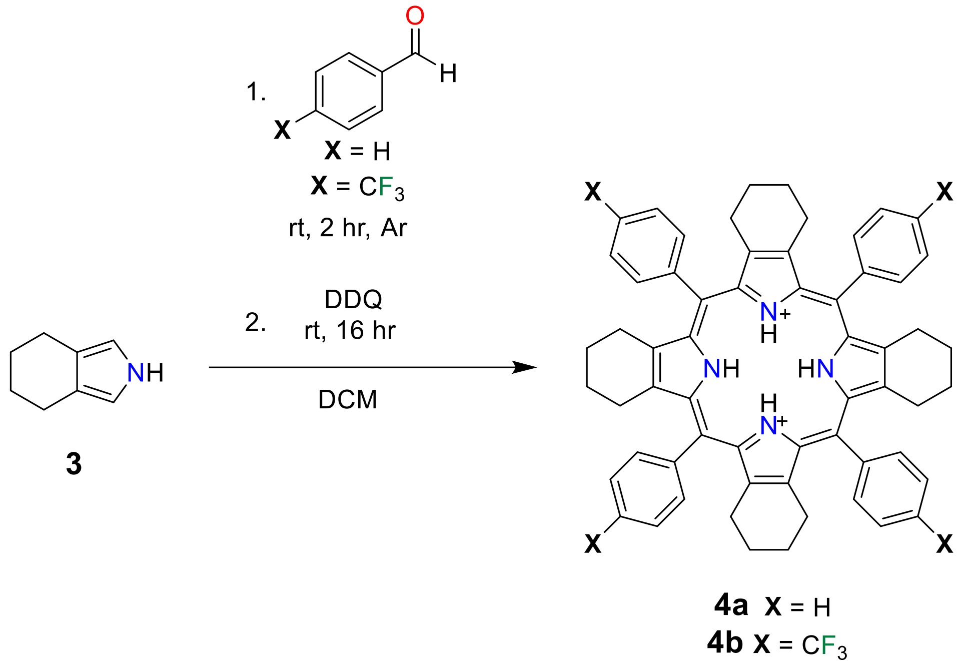

DCM (2.3 L) was added to a three-neck round bottom and bubbled through with argon for one hour. Freshly prepared **3** (2.90 g, 23.9 mmol), from the previous step and the appropriate benzaldehyde (23.9 mmol) were then added and the solution stirred at room temperature for 10 minutes. $BF_3 \cdot OEt_2$ (0.4 mL, 3.3 mmol) was then added and the solution stirred under argon at room temperature for two hours. The resulting deep purple solution was exposed to air and DDQ (5.00 g, 22.0 mmol) was added and the solution stirred at room temperature for 16 hours. The resulting dark green solution was washed with 10% aq. Na_2SO_3 (5 x 500 mL) and 5% aq. HCl and dried over Na_2SO_4. The solvent was reduced in volume to roughly 50 mL by rotary evaporation and then diethyl ether was carefully layered on top of the DCM layer. This was left overnight in a fridge, after which a dark green precipitate was filtered and dried in *vacuo*, to afford a dark green solid **4a** (2.58 g, 3.1 mmol, 52%) or a dark blue green solid **4b** (3.54 g, 3.2 mmol, 54%).

The dark green solid was readily converted to the purple freebase derivative by stirring with NEt_3 in DCM for 30 minutes to deprotonate.

4a (**X** = H) **^{1}H NMR** (400 MHz, $CDCl_3$) δ = 8.40-8.30 (m, 8H, *m*-phenyl-**H**), δ = 7.88-7.75 (d, 12H, *o* and *p*-phenyl-**H**), δ = 2.55-2.40 (m, 8H, cyclohexano-**H**), δ = 2.07-1.92 (m, 8H, cyclohexano-**H**), δ = 1.70-1.55 (m, 8H, cyclohexano-**H**), δ = 1.19-1.00 (m, 8H, cyclohexano-**H**), δ = 0.21 (s, 4H, N**H**).). **HRMS-ASAP(+)** 831.4414 $[M+H]^+$ calculated for $C_{60}H_{55}N_4F_{12}$: 831.4421 (deprotonated **4a**). **UV-Vis** [λ_{max} nm in $CHCl_3$] 471, 618 , 673.

4b (**X** = CF_3) **^{1}H NMR** (400 MHz, $CDCl_3$) δ = 8.52 (d, $^3J_{H-H}$ = 7.7 Hz, 8H, *o*-phenyl-**H**), δ = 8.13 (d, $^3J_{H-H}$ = 7.7 Hz, 8H, *m*-phenyl-**H**), δ = 2.44 (d, $^3J_{H-H}$ = 17.5 Hz, 8H, cyclohexeno-**H**), δ = 1.99 (d, $^3J_{H-H}$ = 17.5 Hz, 8H, cyclohexeno-**H**), δ = 1.75-1.62 (m, 8H, cyclohexeno-**H**), δ = 1.21-1.09 (m, 8H, cyclohexeno-**H**), δ = 0.71 (s, 4H, N**H**). **^{13}C NMR** (125 MHz, $CDCl_3$)), δ = 143.8, (s, α-pyrrole-**C**), δ = 141.2 (s, *i*-phenyl-**C**), δ = 136.7 (s, *o*-phenyl-**C**), δ = 135.5 (s, β-pyrrole-**C**), δ = 131.8 (q, $^2J_{C-F}$ = 32.9 Hz, Hz, *m*-phenyl-**C**), δ = 125.7 (q, $^3J_{C-F}$ = 3.6 Hz, *p*-phenyl-**C**), 124.3 (q, $^1J_{C-F}$ = 273.0 Hz, phenyl-**C**F_3), δ = 116.8 (s, *meso*-**C**), δ = 24,8 (s, cyclohexeno-**C**), δ = 22.4 (s, cyclohexeno-**C**). **^{19}F NMR** (377 MHz, $CDCl_3$) δ = -61.92 (s, 12F, *p*-phenyl-**C**F_3). **HRMS-ASAP(+)** 1103.3917 $[M+H]^+$ calculated for $C_{64}H_{51}N_4F_{12}$: 1103.3874. **UV-Vis** [λ_{max} nm in $CHCl_3$] 465, 614, 674.

4.3.4 Synthesis of Pt4a, Pd4a, Pt4b and Pd4b

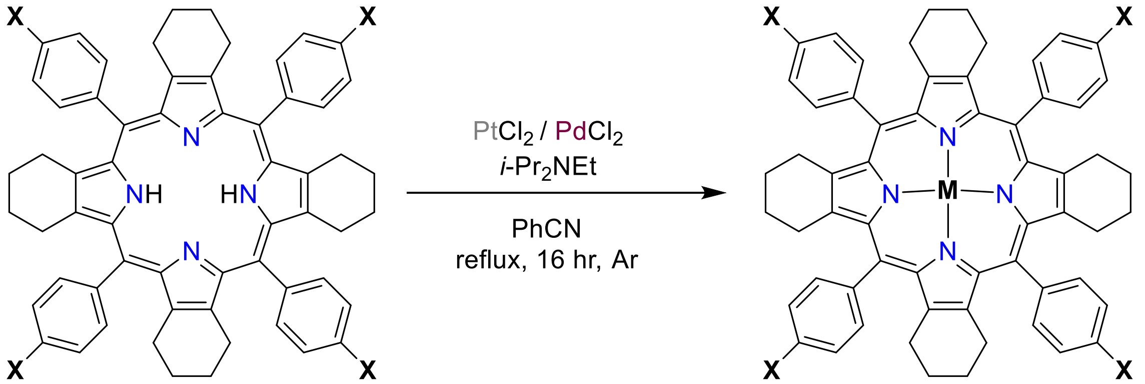

4a X = H
4b X = CF_3

Pt4a M = Pt, **X** = H
Pd4a M = Pd, **X** = H
Pt4b M = Pt, **X** = CF_3
Pd4b M = Pd, **X** = CF_3

$PtCl_2$/$PdCl_2$ (1.72 mmol) was dissolved in PhCN (80 mL) and heated at 120 °C for one hour to form a yellow solution. The freebase derivative of **4a/4b** (0.45 mmol) and *i*-Pr_2NEt (0.3 mL, 1.73 mmol) was then added and the reaction refluxed for 16 hours under a constant supply of argon. The progress of the reaction was monitored by UV-Vis electronic absorption spectroscopy. The solvent was then removed by vacuum distillation and the crude mixture purified by column chromatography (100% $CHCl_3$) to afford red solids **Pt4a/Pd4a/Pt4b/Pd4b** (86-92%).

Pt4a (**M** = Pt, **X** = H) **^{1}H NMR** (500 MHz, $CDCl_3$) δ = 8.06 (d, 8H, $^3J_{H-H}$ = 7.2 Hz, *m*-phenyl-**H**), δ = 7.72-7.60 (m, 12H, *o* and *p*-phenyl-**H**), δ = 2.32 (s, 16H, cyclohexeno-**H**), δ = 1.48 (s, 16H, cyclohexeno-**H**). **HRMS-ASAP(+)** 1024.3907 $[M+H]^+$ calculated for $C_{60}H_{53}N_4Pt$: 1024.3917. **UV-Vis** [λ_{max} nm in $CHCl_3$] 412, 527, 559.

Pd4a (**M** = Pd, **X** = H) **^{1}H NMR** (400 MHz, $CDCl_3$) δ = 8.11 (d, 8H, $^3J_{H-H}$ = 7.1 Hz, *m*-phenyl-**H**), δ = 7.77-7.62 (m, 12H, *o* and *p*-phenyl-**H**), δ = 2.33 (s, 16H, cyclohexeno-**H**), δ = 1.48 (s, 16H, cyclohexeno-**H**). **HRMS-ASAP(+)** 935.3298 $[M+H]^+$ calculated for $C_{60}H_{53}N_4Pd$: 935.3314. **UV-Vis** [λ_{max} nm in $CHCl_3$] 428, 539, 574.

Pt4b (**M** = Pt, **X** = CF_3) **^{1}H NMR** (500 MHz, $CDCl_3$) δ = 8.20 (d, 8H, $^3J_{H-H}$ = 7.8 Hz, phenyl-**H**), δ = 7.77-7.95 (d, 8H, $^3J_{H-H}$ = 7.9 Hz, phenyl-**H**), δ = 2.25 (p, $^3J_{H-H}$ = 3.0 Hz, 16H, cyclohexeno-**H**), δ = 1.49 (p, $^3J_{H-H}$ = 3.2 Hz, 16H, cyclohexeno-**H**). **^{13}C NMR** (125 MHz, $CDCl_3$) δ = 144.7 (s, *i*-phenyl-**C**), δ = 140.2 (s, α-pyrrole-**C**), δ = 139.8 (s, β-pyrrole-**C**), δ = 134.4 (s, *o*-phenyl-**C**), δ = 130.8 (q, $^2J_{C-F}$ = 30.8 Hz, Hz, *m*-phenyl-**C**), δ = 124.7 (q, $^3J_{C-F}$ = 3.9 Hz, *p*-phenyl-**C**), 124.6 (q, $^1J_{C-F}$ = 273.1 Hz, phenyl-**C**F_3), δ = 118.3 (s, *meso*-**C**), δ = 26.7 (s, cyclohexeno-**C**), δ = 23.6 (s, cyclohexeno-**C**). **^{19}F NMR** (471 MHz, $CDCl_3$) δ = -61.89 (s, 12F, phenyl-C**F_3**). **HRMS-ASAP(+)** 1296.3339 $[M+H]^+$ calculated for $C_{64}H_{49}F_{12}N_4Pt$: 1296.3413. **UV-Vis** [λ_{max} nm in $CHCl_3$] 412 (Soret band), 527, 562 (Q bands).

Pd4b (**M** = Pd, **X** = CF_3) **^{1}H NMR** (500 MHz, $CDCl_3$) δ = 8.22 (d, 8H, $^3J_{H-H}$ = 7.7 Hz, phenyl-**H**), δ = 7.77-7.96 (d, 8H, $^3J_{H-H}$ = 7.7 Hz, phenyl-**H**), δ = 2.24 (p, $^3J_{H-H}$ = 3.1 Hz, 16H, cyclohexeno-**H**), δ = 1.48 (p, $^3J_{H-H}$ = 3.0 Hz, 16H, cyclohexeno-**H**). **^{13}C NMR** (125 MHz, $CDCl_3$) δ = 144.9 (s, *i*-phenyl-**C**), δ = 142.4 (s, α-pyrrole-**C**), δ = 140.7 (s, β-pyrrole-**C**), δ = 134.5 (s, *o*-phenyl-**C**), δ = 130.8 (q, $^2J_{C-F}$ = 32.6 Hz, Hz, *m*-phenyl-**C**), δ = 124.8 (q, $^3J_{C-F}$ = 3.5 Hz, *p*-phenyl-**C**), 124.6 (q, $^1J_{C-F}$ = 272.9 Hz, phenyl-**C**F_3), δ = 118.3 (s, *meso*-**C**), δ = 26.6 (s, cyclohexeno-**C**), δ = 23.5 (s, cyclohexeno-**C**). **^{19}F NMR** (471 MHz, $CDCl_3$) δ = -61.89 (s, 12F, phenyl-C**F_3**). **HRMS-ASAP(+)** 1207.2808 $[M+H]^+$ calculated for $C_{64}H_{49}F_{12}N_4Pd$: 1207.2816. **UV-Vis** [λ_{max} nm in $CHCl_3$] 428 (Soret band), 542, 577 (Q bands).

4.3.5 Synthesis of PtBP, PdBP, Pt-*p*CF_3-BP and Pd-*p*CF_3-BP

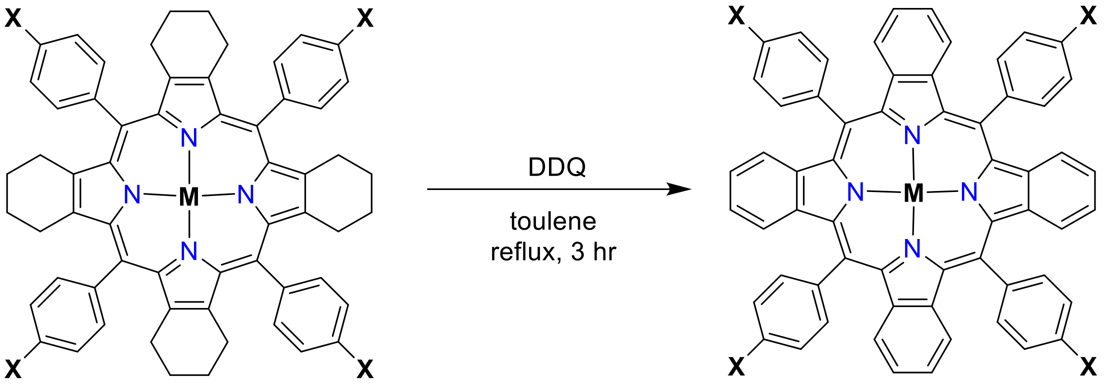

**Pt4a** **M** = Pt, **X** = H	**PtBP** **M** = Pt, **X** = H
**Pd4a** **M** = Pd, **X** = H	**PdBP** **M** = Pd, **X** = H
**Pt4b** **M** = Pt, **X** = CF_3	**Pt-*p*CF_3-BP** **M** = Pt, **X** = CF_3
**Pd4b** **M** = Pd, **X** = CF_3	**Pt-*p*CF_3-BP** **M** = Pd, **X** = CF_3

Pt4a/Pd4a/Pt4b/Pd4b (0.20 mmol) was dissolved in toluene (150 mL) and heated to 80 °C. DDQ (0.54 g, 2.4 mmol) was then added, forming a dark green solution, and the reaction refluxed for 3 hours. The progress of the reaction was monitored by UV-Vis electronic absorption spectroscopy. Upon completion, the brown solution was washed with 10% aq. Na_2SO_3 (3 x 50 mL), the isolated green organic layer was dried over Na_2SO_4 and then the solvent removed by rotary evaporation. The crude blue/green product was first purified by column chromatography (100% $CHCl_3$). Then the blue/green residue was dissolved in chloroform, hexane carefully layered on top and left to stand in a fridge overnight. The resulting shiny purple crystals were filtered and washed with hexane to afford **PtBP** (60.5 mg, 0.06 mmol, 29%), **PdBP** (128.7 mg, 0.14 mmol, 70%), **Pt-*p*CF_3-BP** (76.8 mg, 0.06 mmol, 30%), **Pd-*p*CF_3-BP** (190.6 mg, 0.16 mmol, 81%).

PtBP (**M** = Pt, **X** = H) **^{1}H NMR** (500 MHz, $CDCl_3$) δ = 8.26 (d, 8H, $^3J_{H\text{-}H}$ = 7.9 Hz, *o*-phenyl-**H**), δ = 7.93 (tt, 4H, $^3J_{H\text{-}H}$ = 7.7 Hz, $^4J_{H\text{-}H}$ = 1.4 Hz, *p*-phenyl-**H**), δ = 7.86 (tt, 8H, $^3J_{H\text{-}H}$ = 7.7 Hz, $^4J_{H\text{-}H}$ = 1.4 Hz, *m*-phenyl-**H**), δ = 7.22 (q, 8H, $^3J_{H\text{-}H}$ = 3.1 Hz, benzo-**H**), δ = 7.08 (q, 8H, $^3J_{H\text{-}H}$ = 3.2 Hz, benzo-**H**). **^{13}C NMR** (125 MHz, $CDCl_3$) δ = 141.8 (s, *i*-phenyl-**C**), δ = 137.7 (s, β-pyrrole-**C**), δ = 133.8 (s, *o*-phenyl-**C**), δ = 129.4 (s, *m*-phenyl-**C**), δ = 129.3 (s, *p*-phenyl-**C**), δ = 125.5 (s, benzo-**C**), δ = 124.2 (s, benzo-**C**), δ = 118.6 (s, HMBC, *meso*-C). **HRMS-ASAP(+)** 1008.2649 $[M+H]^+$ calculated for $C_{60}H_{37}N_4Pt$: 1008.2665. **UV-Vis** [λ_{max} nm in $CHCl_3$] 430 (Soret band), 564, 614 (Q bands).

PdBP (**M** = Pd, **X** = H) **^{1}H NMR** (400 MHz, $CDCl_3$) δ = 8.27 (d, 8H, $^3J_{H\text{-}H}$ = 7.9 Hz, *o*-phenyl-**H**), δ = 7.93 (tt, 4H, $^3J_{H\text{-}H}$ = 7.7 Hz, $^4J_{H\text{-}H}$ = 1.4 Hz, *p*-phenyl-**H**), δ = 7.85 (tt, 8H, $^3J_{H\text{-}H}$ = 7.7 Hz, $^4J_{H\text{-}H}$ = 1.4 Hz, *m*-phenyl-**H**), δ = 7.21 (q, 8H, $^3J_{H\text{-}H}$ = 3.1 Hz, benzo-**H**), δ = 7.11 (q, 8H, $^3J_{H\text{-}H}$ = 3.2 Hz, benzo-**H**). **^{13}C NMR** (125 MHz,

$CDCl_3$) δ = 142.0 (s, *i*-phenyl-**C**), δ = 138.6 (s, α-pyrrole-**C**), δ = 138.1 (s, β-pyrrole-**C**), δ = 134.1 (s, *o*-phenyl-**C**), δ = 129.3 (s, *m*-phenyl-**C**), δ = 129.3 (s, *p*-phenyl-**C**), δ = 125.4 (s, benzo-**C**), δ = 124.0 (s, benzo-**C**), δ = 118.2 (s, *meso*-**C**). **HRMS-ASAP(+)** 919.2082 $[M+H]^+$ calculated for $C_{60}H_{37}N_4Pd$: 919.2048. **UV-Vis** [λ_{max} nm in $CHCl_3$] 442 (Soret band), 577, 629 (Q bands).

Pt-*p*CF$_3$-BP (**M** = Pt, **X** = CF_3) **^{1}H NMR** (500 MHz, $CDCl_3$) δ = 8.43 (d, 8H, $^3J_{H-H}$ = 7.9 Hz, *o*-phenyl-**H**), δ = 8.17 (d, 8H, $^3J_{H-H}$ = 7.9 Hz, *m*-phenyl-**H**), δ = 7.26 (q, 8H, $^3J_{H-H}$ = 3.0 Hz, benzo-**H**), δ = 6.99 (q, 8H, $^3J_{H-H}$ = 3.1 Hz, benzo-**H**). **^{13}C NMR** (125 MHz, $CDCl_3$) δ = 145.0 (s, *i*-phenyl-**C**), 137.3 (s, β-pyrrole-**C**), 136.1 (s, α-pyrrole-**C**), δ = 134.3 (s, *o*-phenyl-**C**), δ = 131.8 (q, $^2J_{C-F}$ = 32.5 Hz, *m*-phenyl-**C**), δ = 126.4 (q, $^3J_{C-F}$ = 3.6 Hz, *m*-phenyl-**C**), δ = 126.2 (s, benzo-**C**), δ = 126.0 (q, HMBC, $^1J_{C-F}$ = 270.5 Hz, phenyl-**C**F_3) δ = 124.0 (s, benzo-**C**), δ = 117.4 (s, *meso*-**C**). **^{19}F NMR** (471 MHz, $CDCl_3$) δ = -61.81 (s, 12F, phenyl-C**F$_3$**). **HRMS-ASAP(+)** 1280.2129 $[M+H]^+$ calculated for $C_{64}H_{33}F_{12}N_4Pt$: 1280.2161. **UV-Vis** [λ_{max} nm in $CHCl_3$] 427 (Soret band), 567, 618 (Q bands).

Pd-*p*CF$_3$-BP (**M** = Pd, **X** = CF_3) **^{1}H NMR** (500 MHz, $CDCl_3$) δ = 8.43 (d, 8H, $^3J_{H-H}$ = 7.8 Hz, *o*-phenyl-**H**), δ = 8.19 (d, 8H, $^3J_{H-H}$ = 7.8 Hz, *m*-phenyl-**H**), δ = 7.26 (q, 8H, $^3J_{H-H}$ = 3.1 Hz, benzo-**H**), δ = 7.03 (q, 8H, $^3J_{H-H}$ = 3.1 Hz, benzo-**H**). **^{13}C NMR** (125 MHz, $CDCl_3$) δ = 145.1 (s, *i*-phenyl-**C**), 138.5 (s, α-pyrrole-**C**), δ = 137.8 (s, β-pyrrole-**C**), δ = 134.5 (s, *o*-phenyl-**C**), δ = 131.7 (q, $^2J_{C-F}$ = 34.5 Hz, *p*-phenyl-**C**), δ = 126.4 (q, $^3J_{C-F}$ = 3.6 Hz, *m*-phenyl-**C**), δ = 126.1 (s, benzo-**C**), δ = 124.6 (q, HMBC, $^1J_{C-F}$ = 271.3 Hz, phenyl-**C**F_3), δ = 123.8 (s, benzo-**C**), δ = 117.0 (s, *meso*-C). **^{19}F NMR** (471 MHz, $CDCl_3$) δ = -61.79 (s, 12F, phenyl-C**F$_3$**). **HRMS-ASAP(+)** 1191.1566 $[M+H]^+$ calculated for $C_{64}H_{33}F_{12}N_4Pd$: 1191.1564. **UV-Vis** [λ_{max} nm in $CHCl_3$] 440 (Soret band), 582, 631 (Q bands).

4.4 Characterisation data

4.4.1 Characterisation data of 2

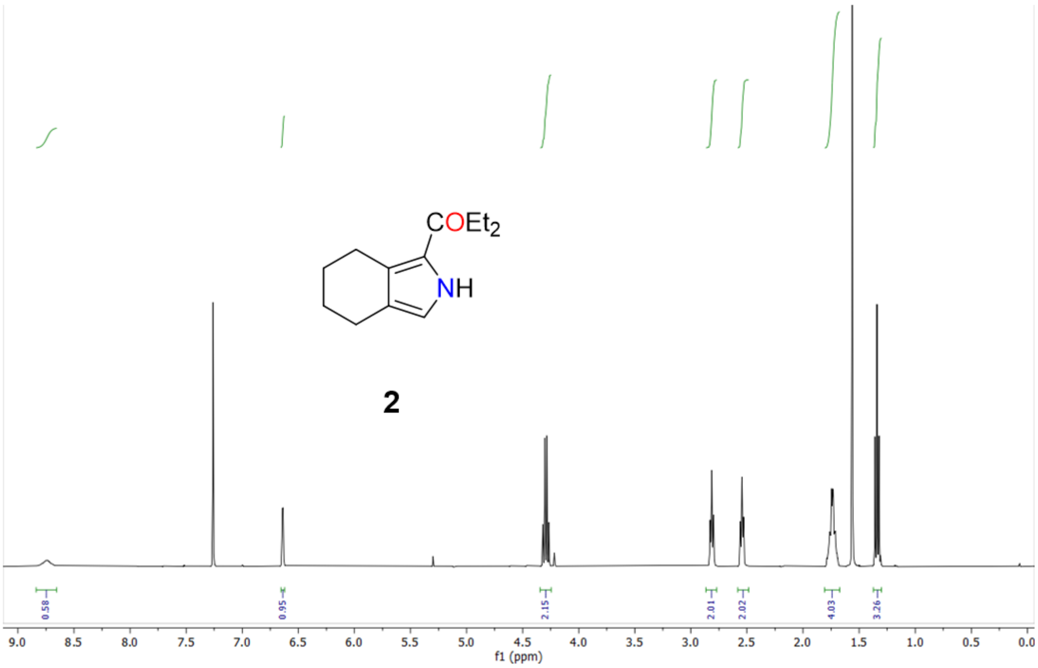

Figure S1. ^{1}H NMR spectrum of **2** in $CDCl_3$.

4.4.2 Characterisation data of 4a

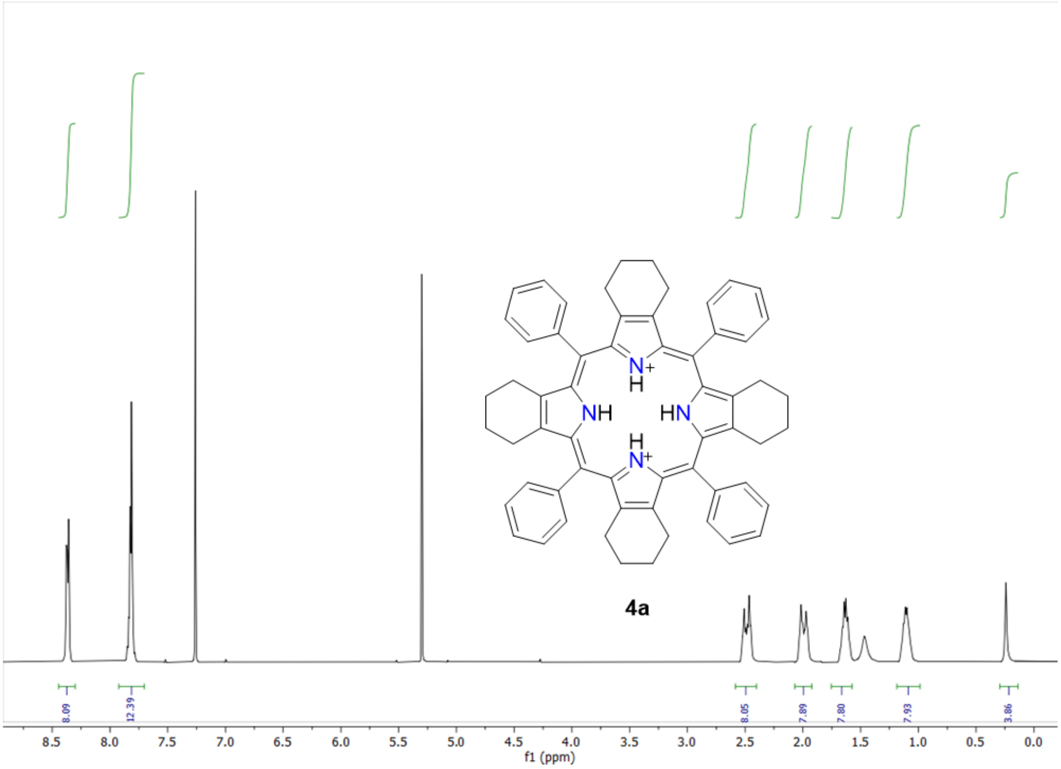

Figure S2. ^{1}H NMR spectrum of **4a** in $CDCl_3$.

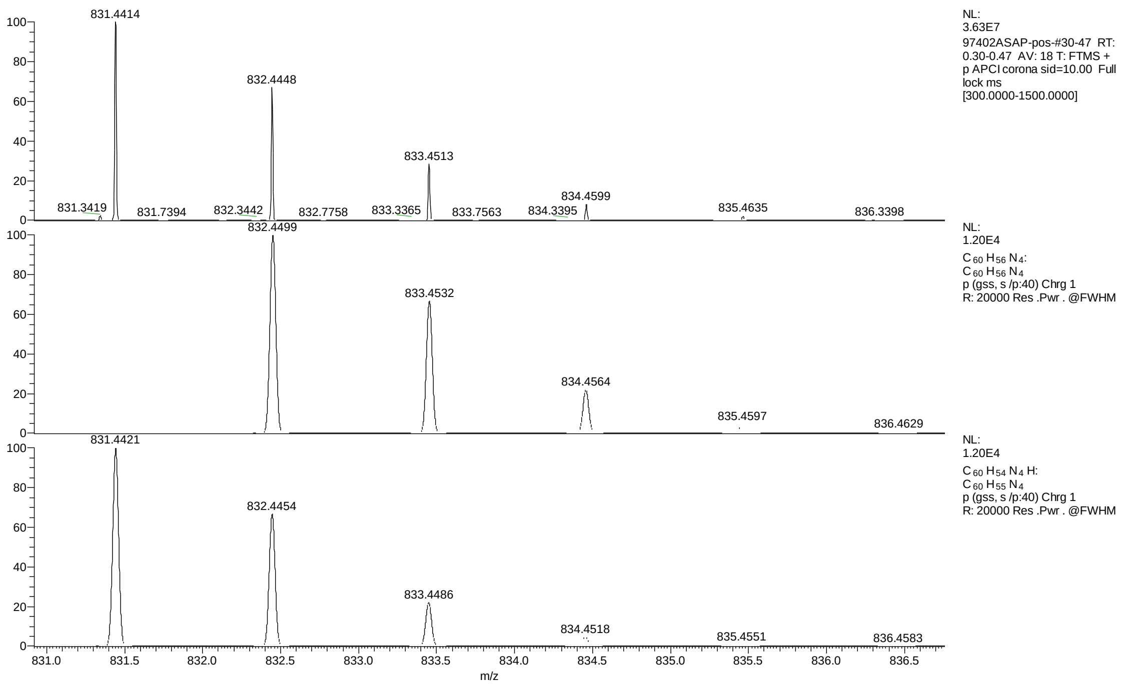

Figure S3. HRMS-ASAP(+) of **4a**.

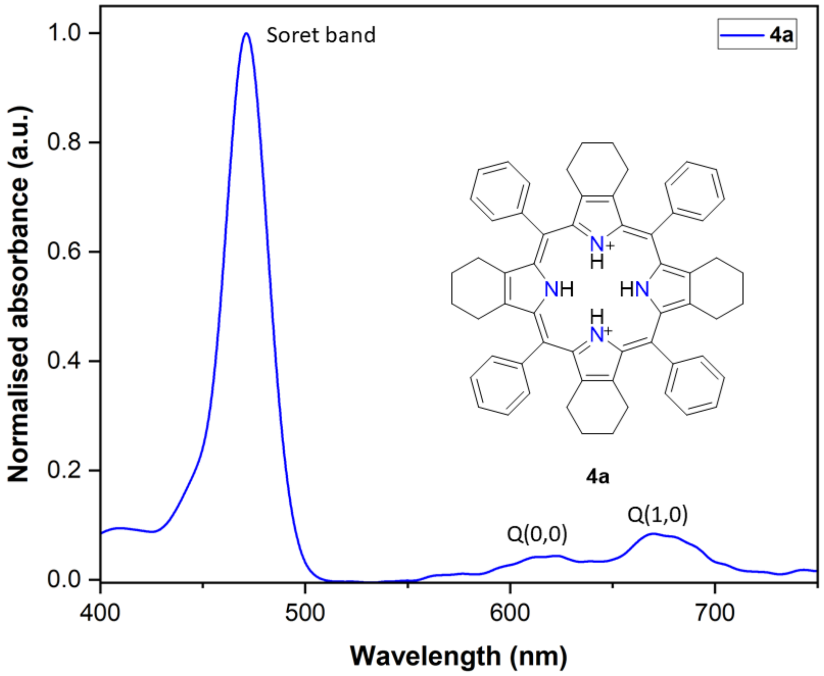

Figure S4. The UV-Vis electronic absorption spectrum of **4a** in chloroform at a concentration of 2 µM.

4.4.3 Characterisation data of 4b

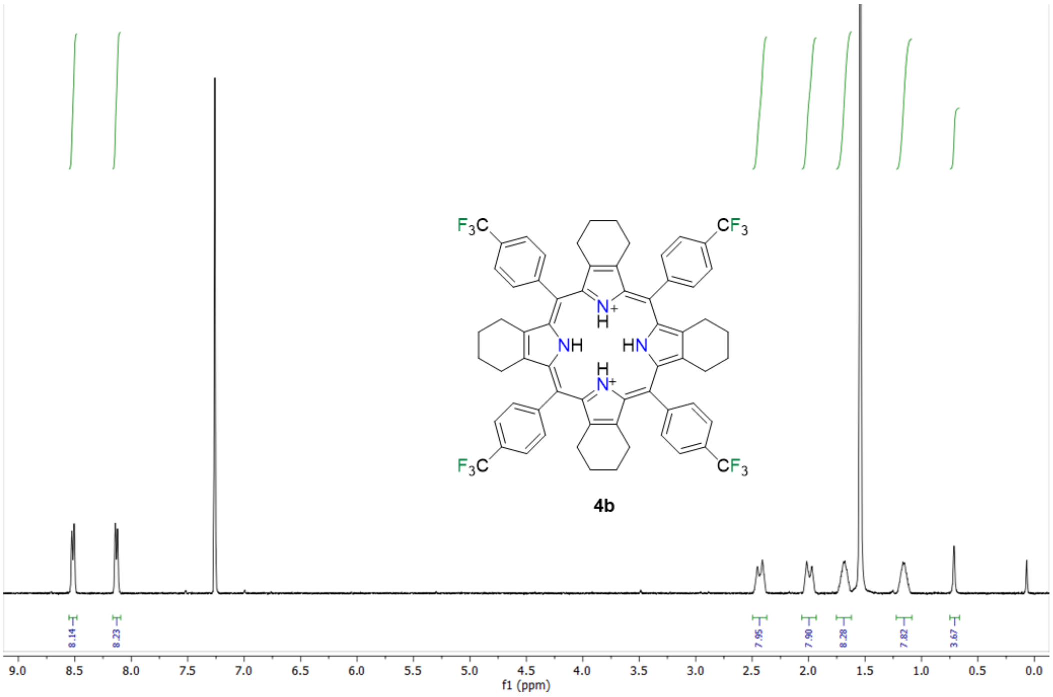

Figure S5. ^{1}H NMR spectrum of **4b** in $CDCl_3$.

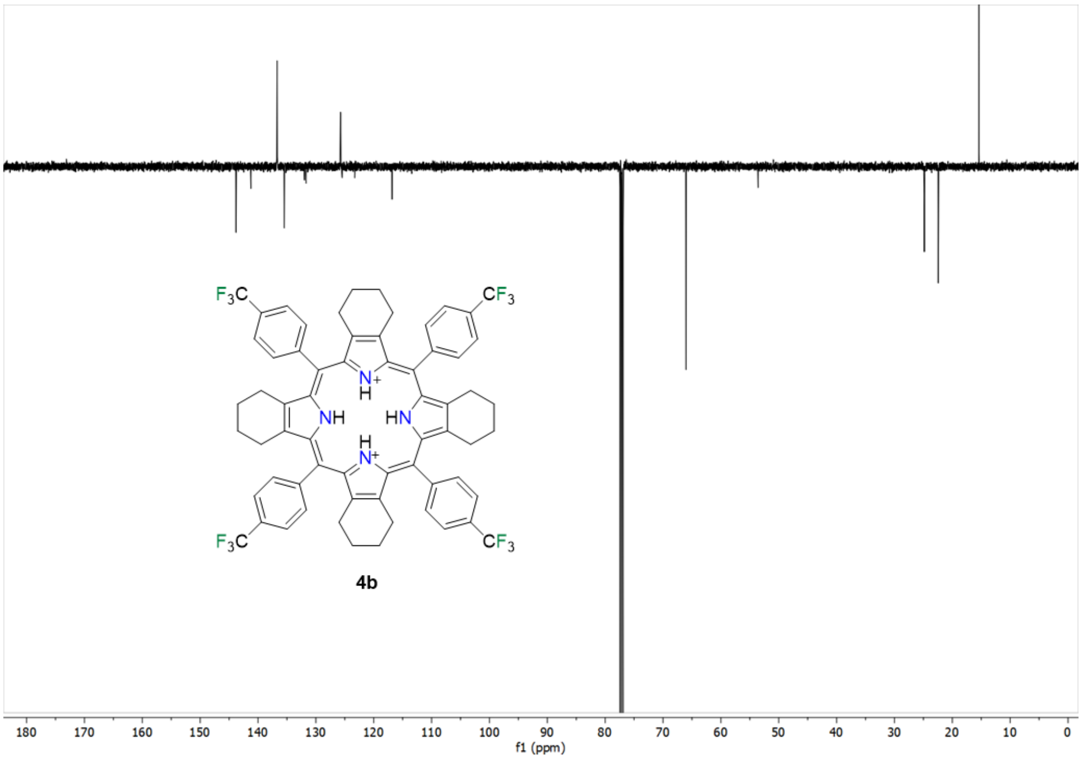

Figure S6. $^{13}C\{^{1}H\}$ DEPTQ NMR spectrum of **4b** in $CDCl_3$.

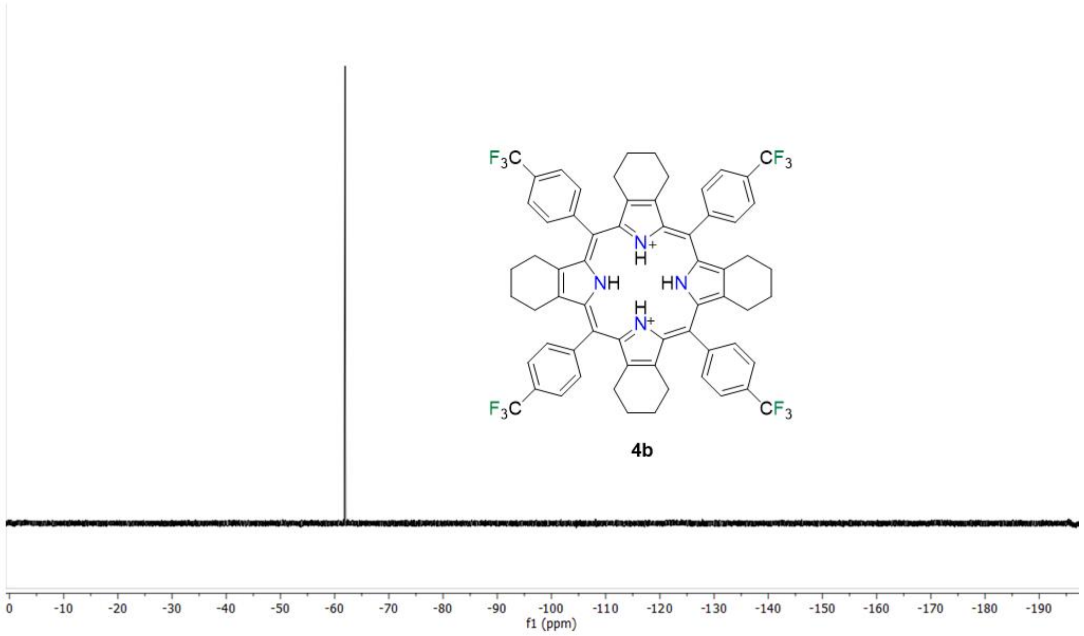

Figure S7. ^{19}F NMR spectrum of **4b** in $CDCl_3$.

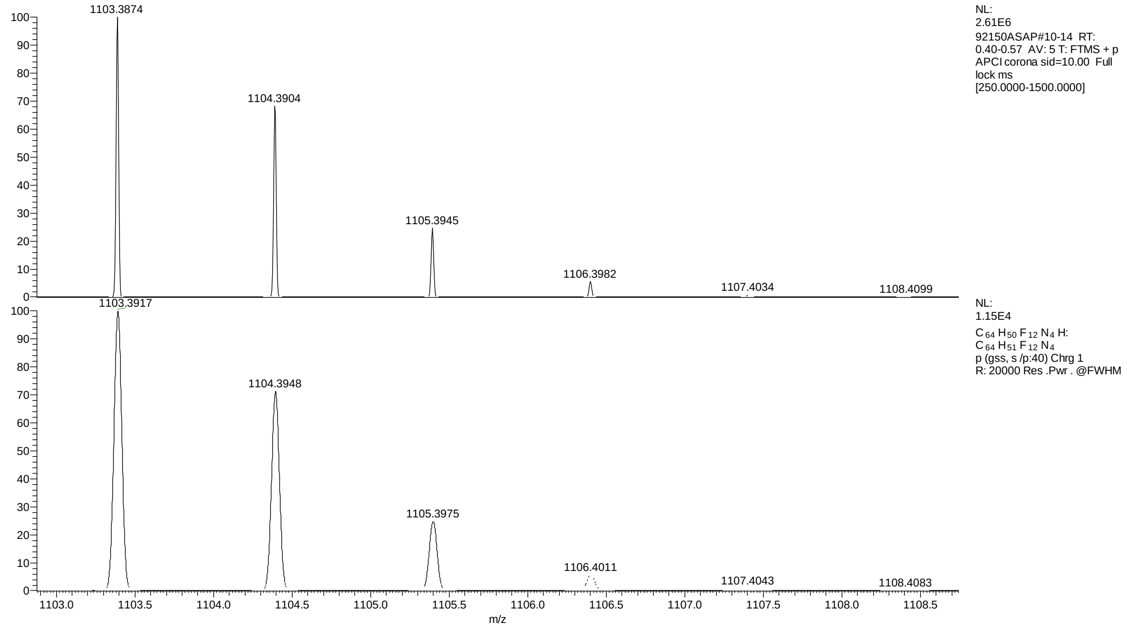

Figure S8. HRMS-ASAP(+) spectrum of **4b**.

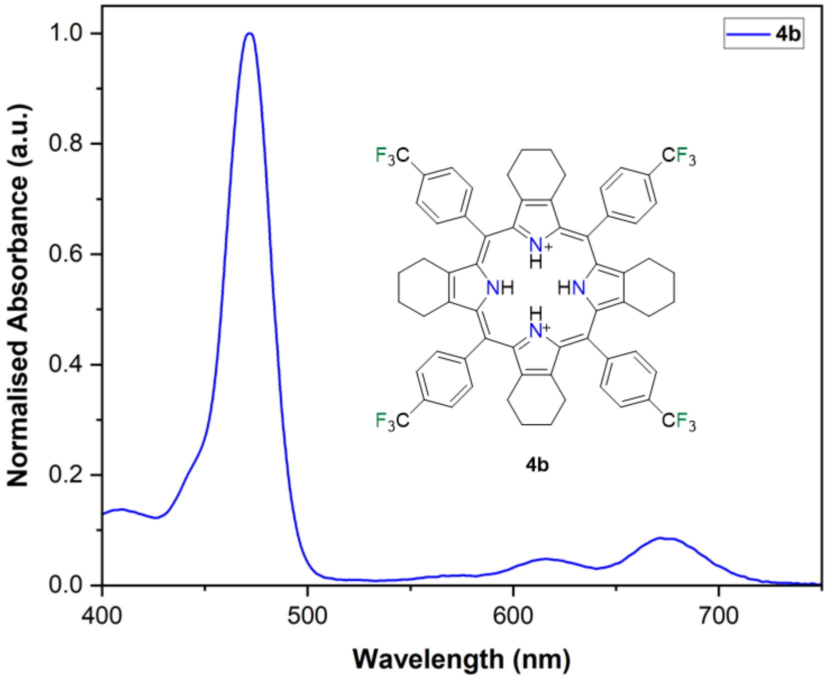

Figure S9. The UV-Vis electronic absorption spectrum of **4b** in chloroform at a concentration of 2 μM.

4.4.4 Characterisation data of Pt4a

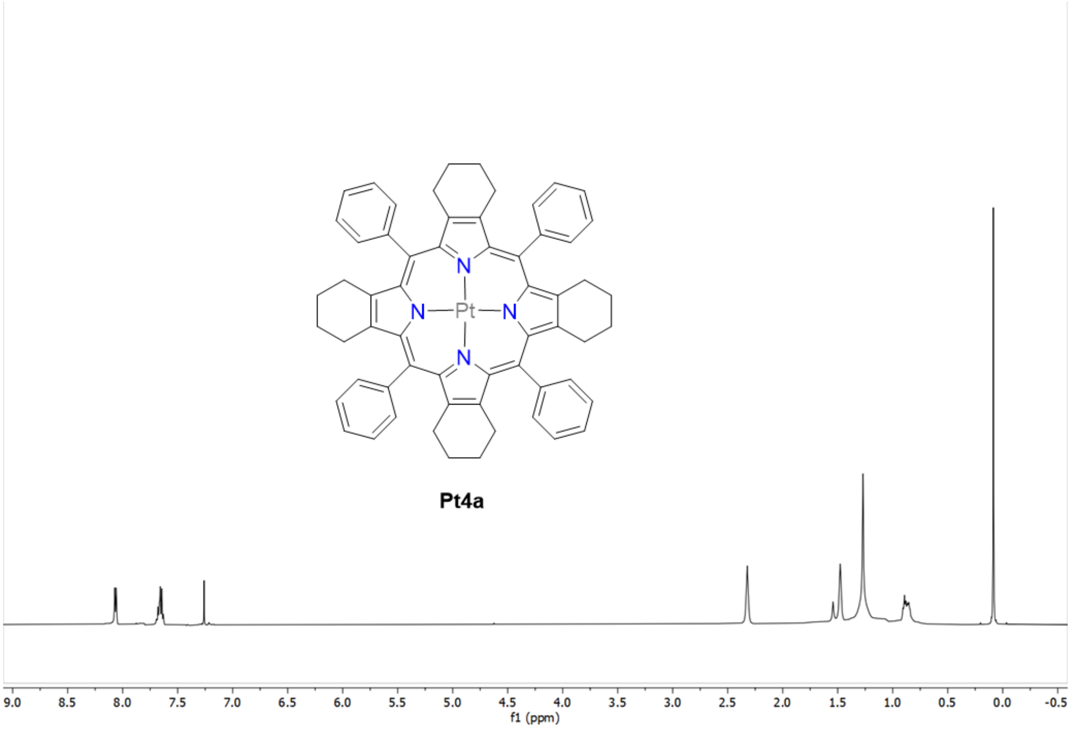

Figure S10. ^{1}H NMR spectrum of **Pt4a** in $CDCl_3$.

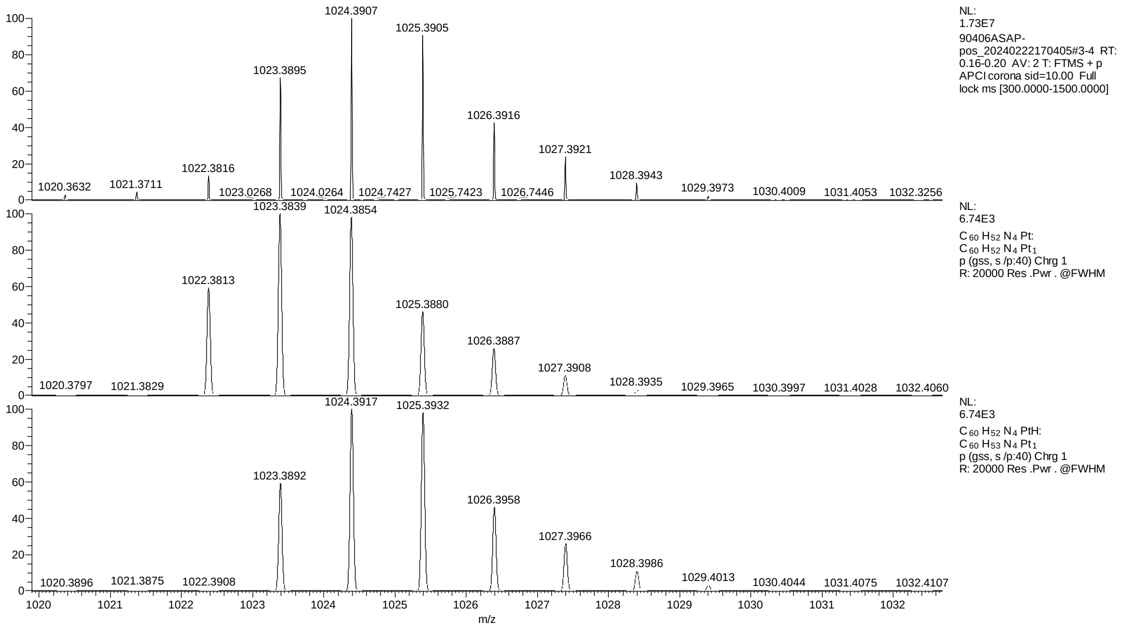

Figure S11. HRMS-ASAP(+) spectrum of **Pt4a**

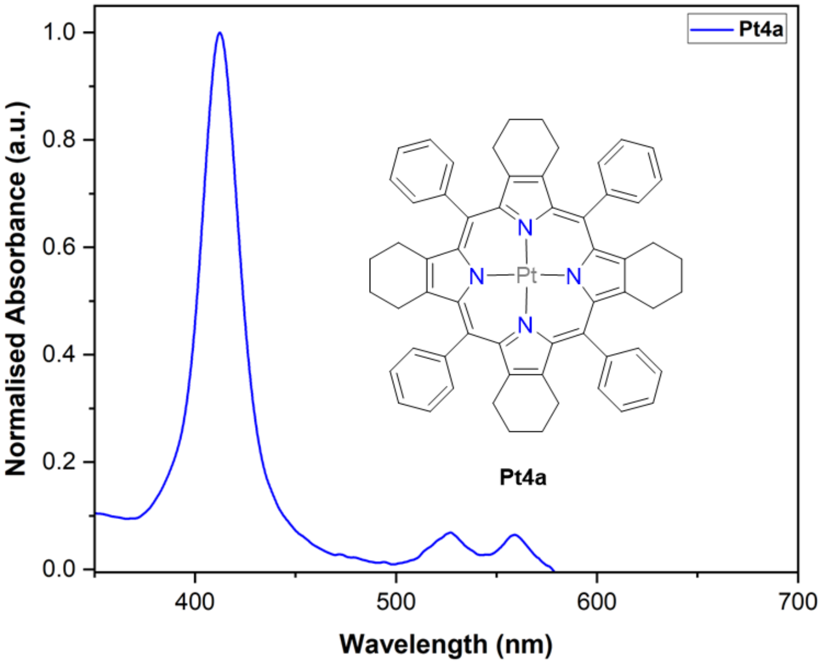

Figure S12. The UV-Vis electronic absorption spectrum of **Pt4a** in chloroform at a concentration of 2 μM.

4.4.5 Characterisation data of Pd4a

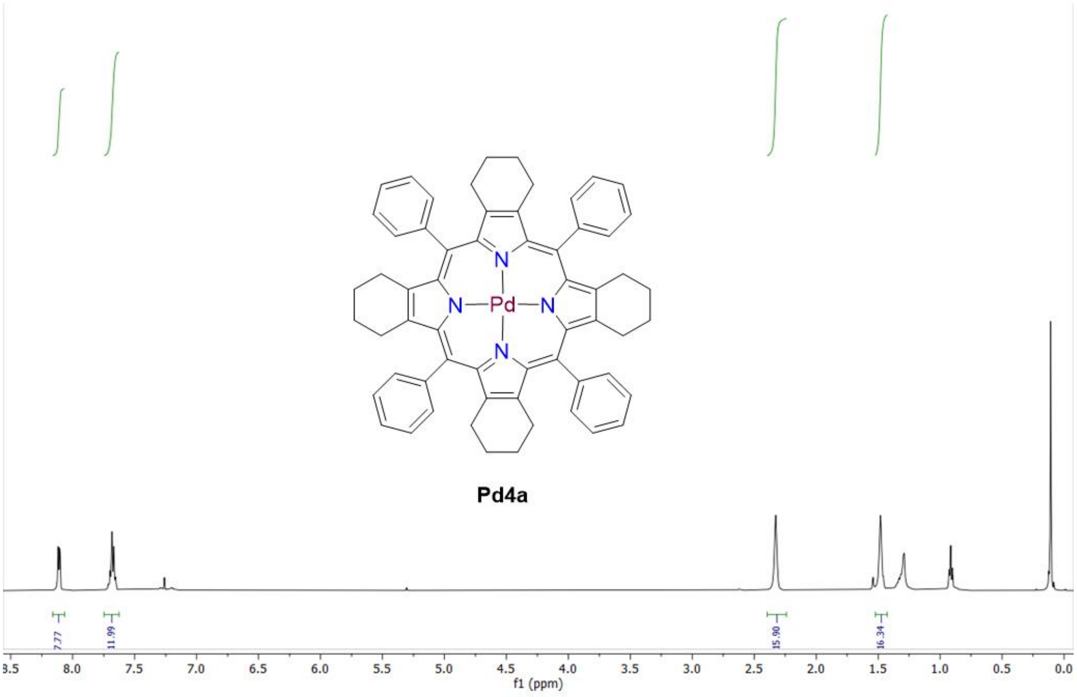

Figure S13. ^{1}H NMR spectrum of **Pd4a** in $CDCl_3$.

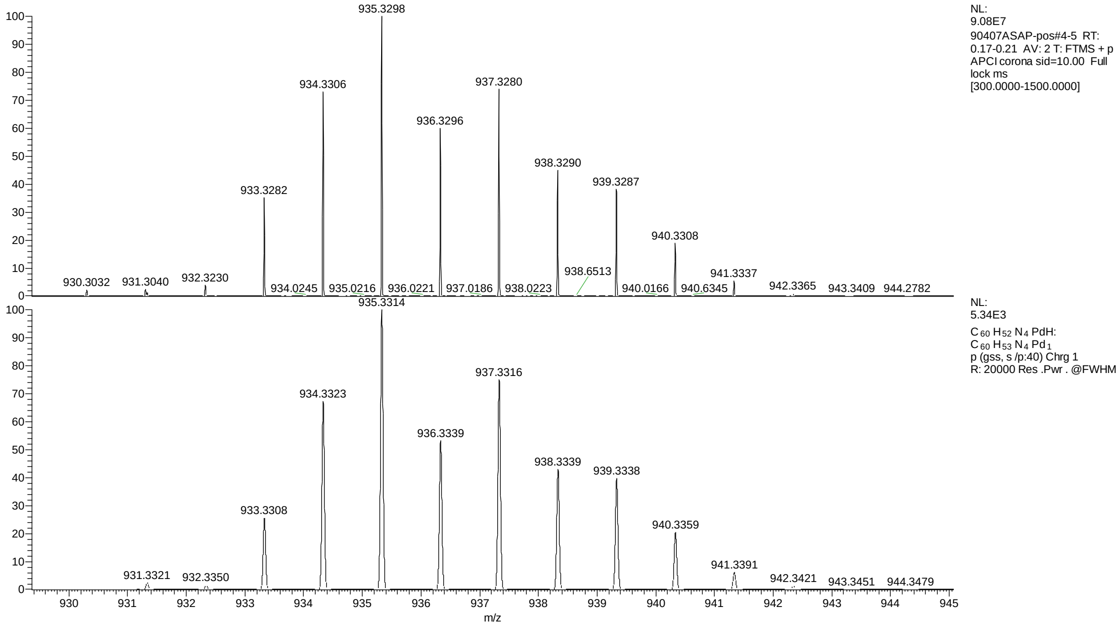

Figure S14. HRMS-ASAP(+) spectrum of **Pd4a**.

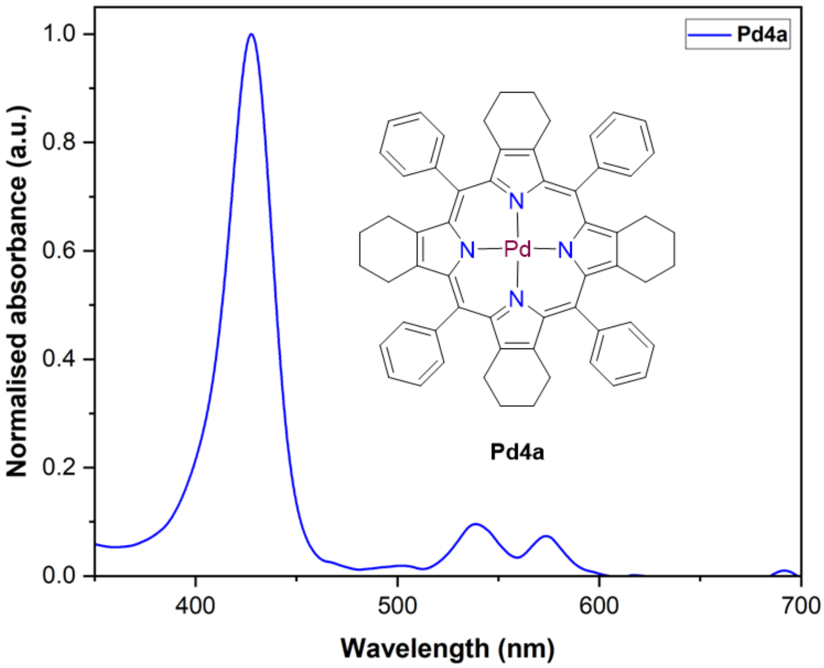

Figure S15. The UV-Vis electronic absorption spectrum of **Pd4a** in chloroform at a concentration of 2 μM.

4.4.6 Characterisation data of Pt4b

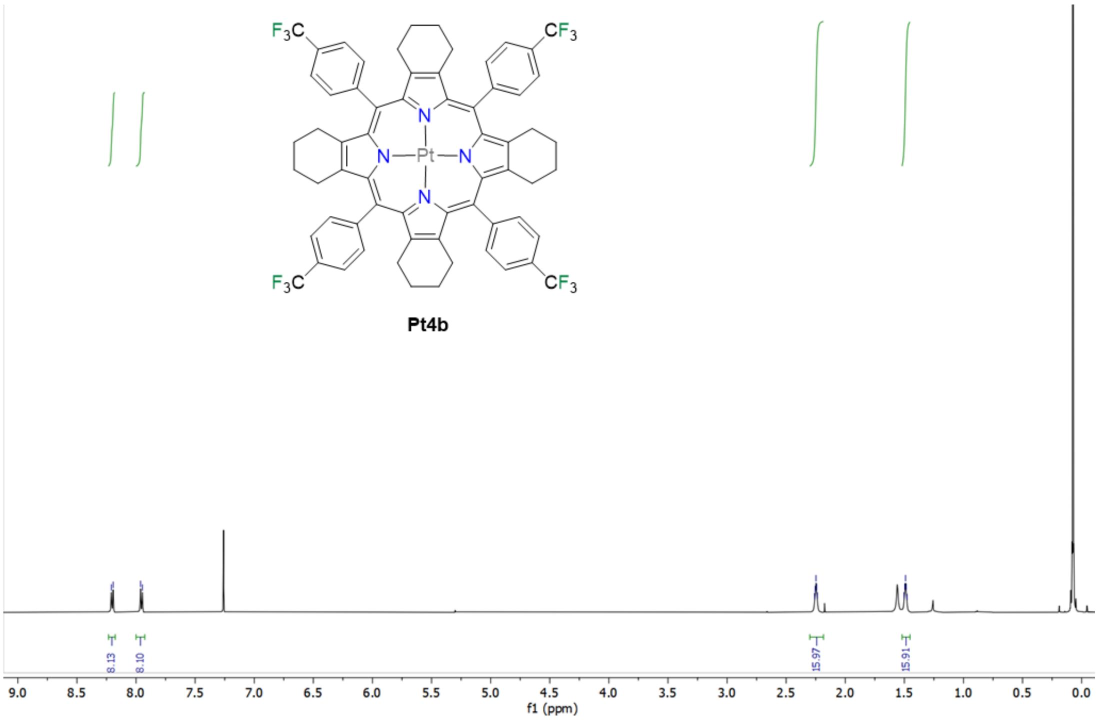

Figure S16. ^{1}H NMR spectrum of **Pt4b** in $CDCl_3$.

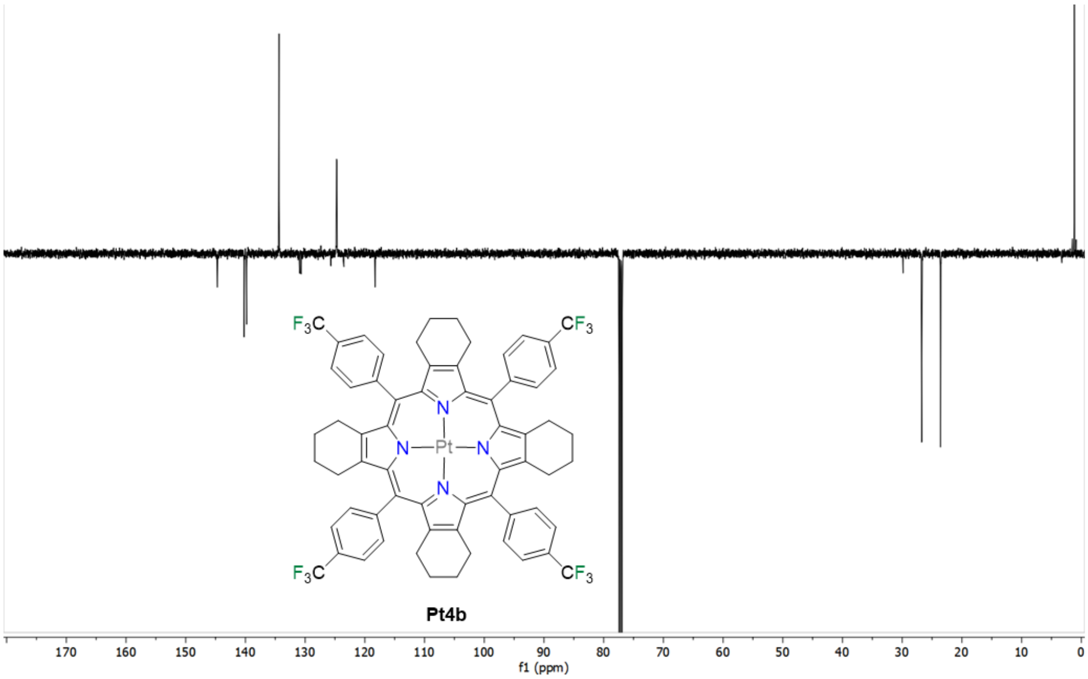

Figure S17. $^{13}C\{^{1}H\}$ DEPTQ NMR spectrum of **Pt4b** in $CDCl_3$.

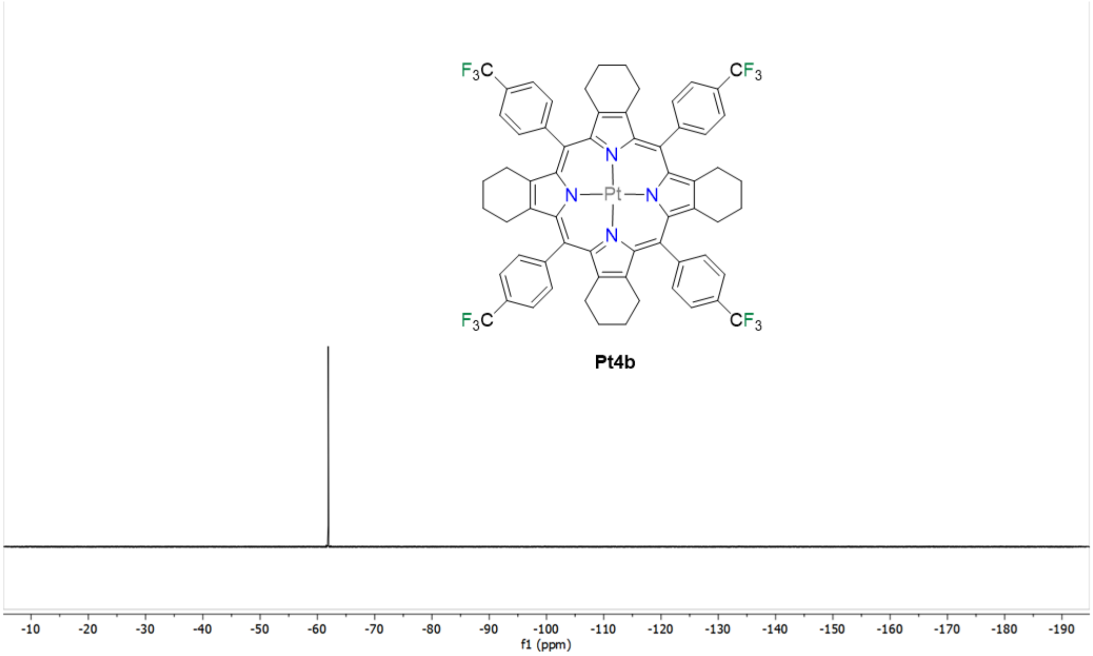

Figure S18. ^{19}F NMR spectrum of **Pt4b** in $CDCl_3$.

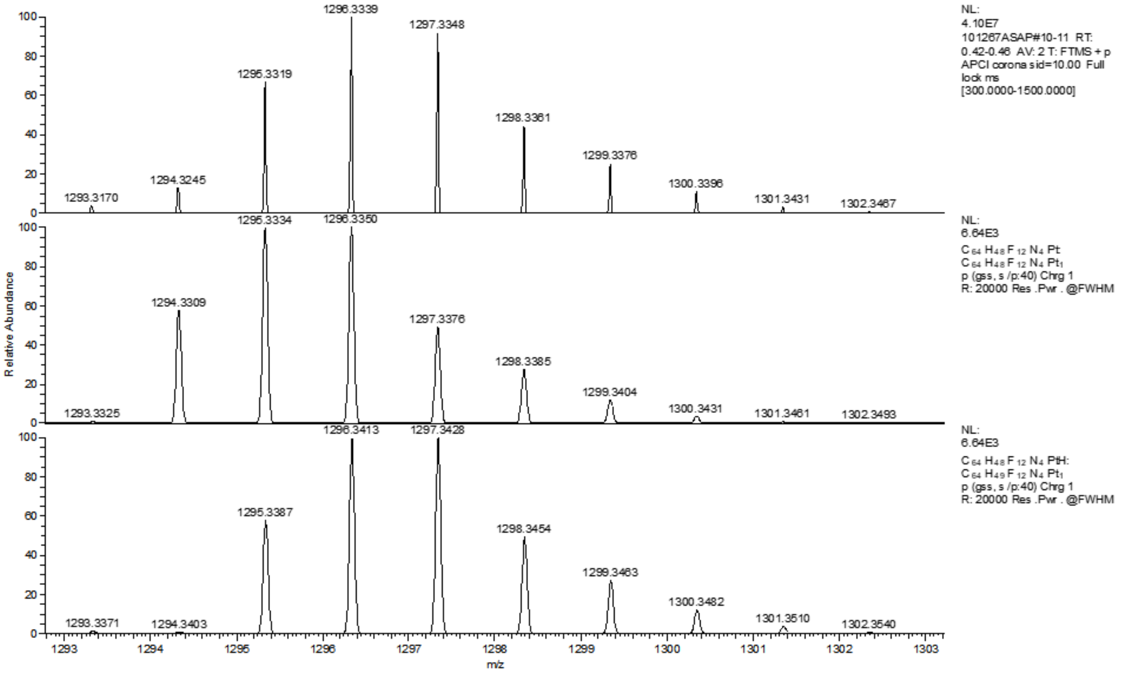

Figure S19. HRMS-ASAP(+) spectrum of **Pt4b**

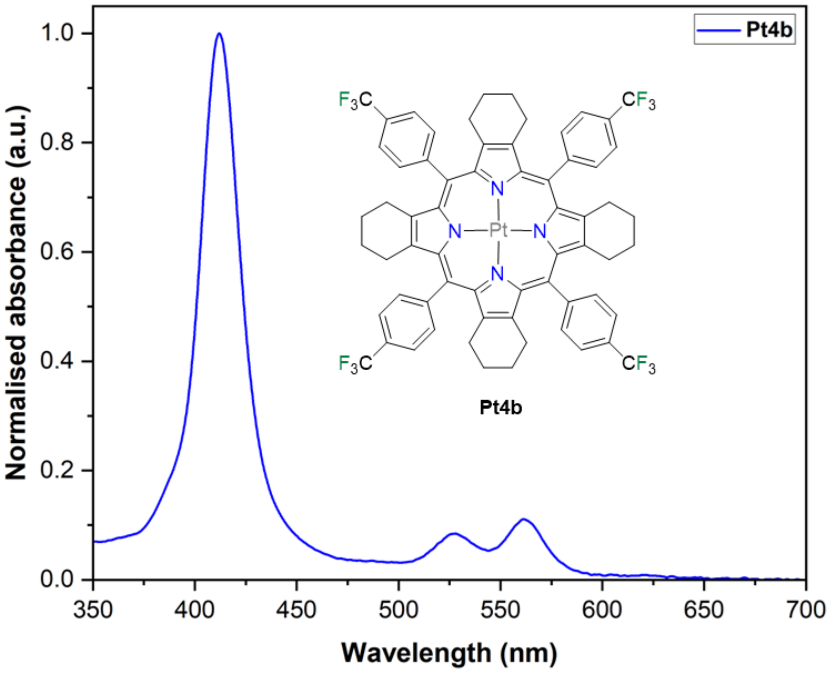

Figure S20. The UV-Vis electronic absorption spectrum of **Pt4b** in chloroform at a concentration of 2 μM.

4.4.7 Characterisation data of Pd4b

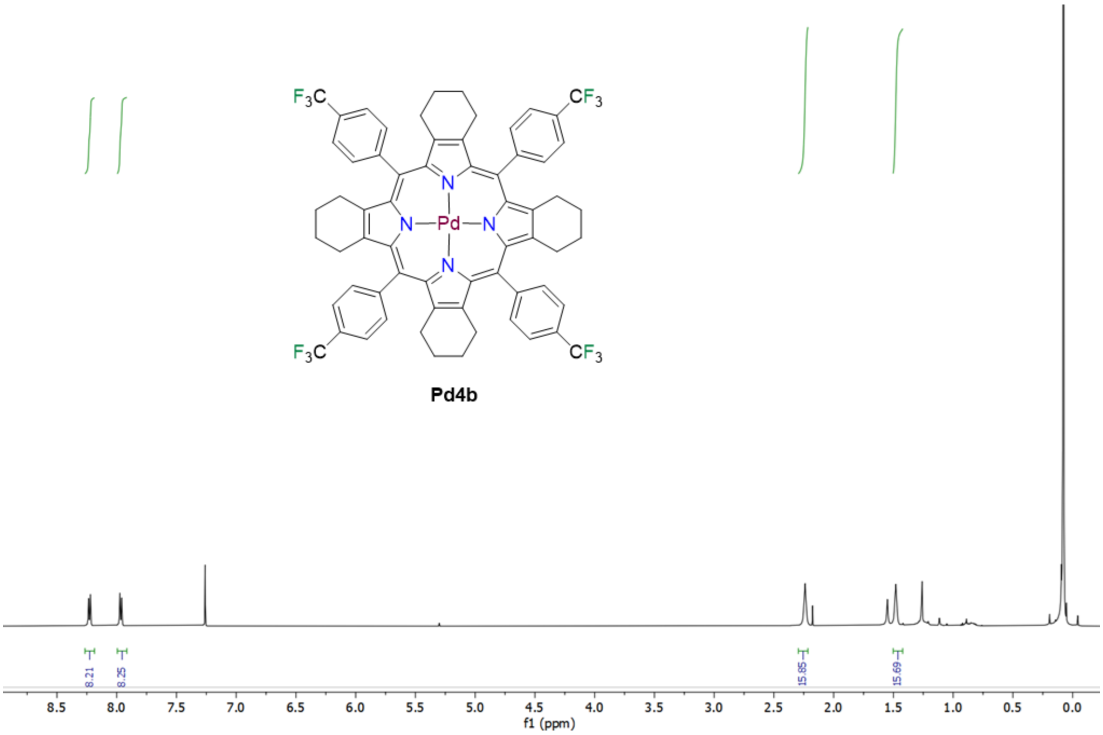

Figure S21. ^{1}H NMR spectrum of **Pd4b** in $CDCl_3$.

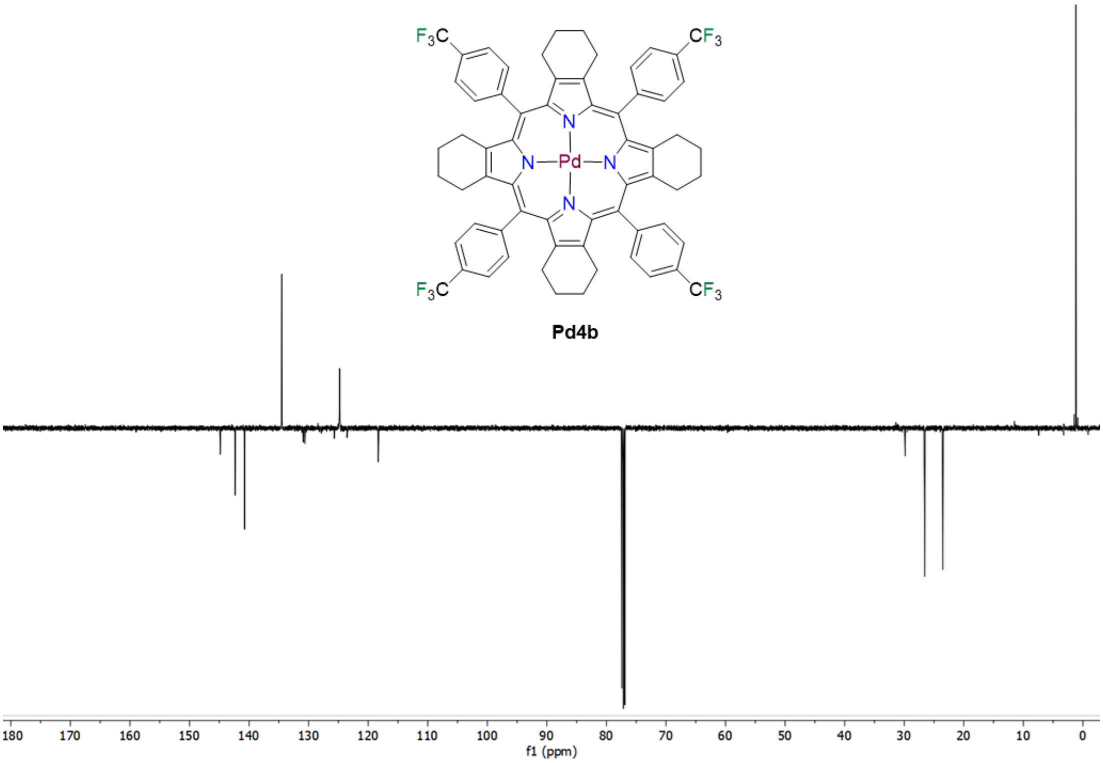

Figure S22. $^{13}C\{^{1}H\}$ DEPTQ NMR spectrum of **Pd4b** in $CDCl_3$.

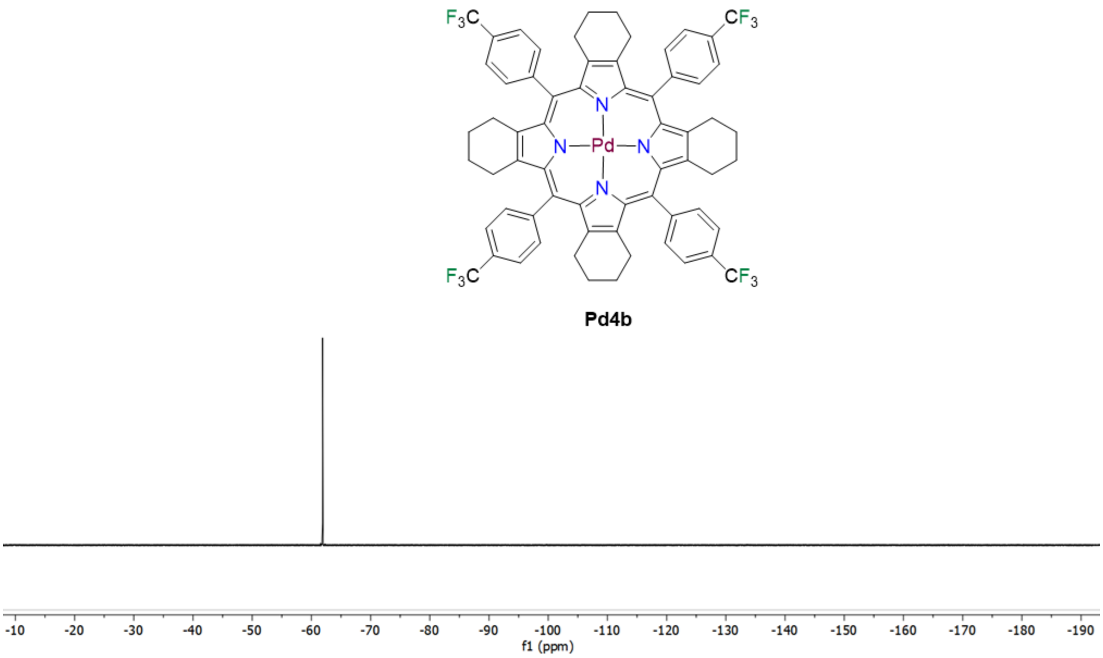

Figure S23. ^{19}F NMR spectrum of **Pd4b** in $CDCl_3$.

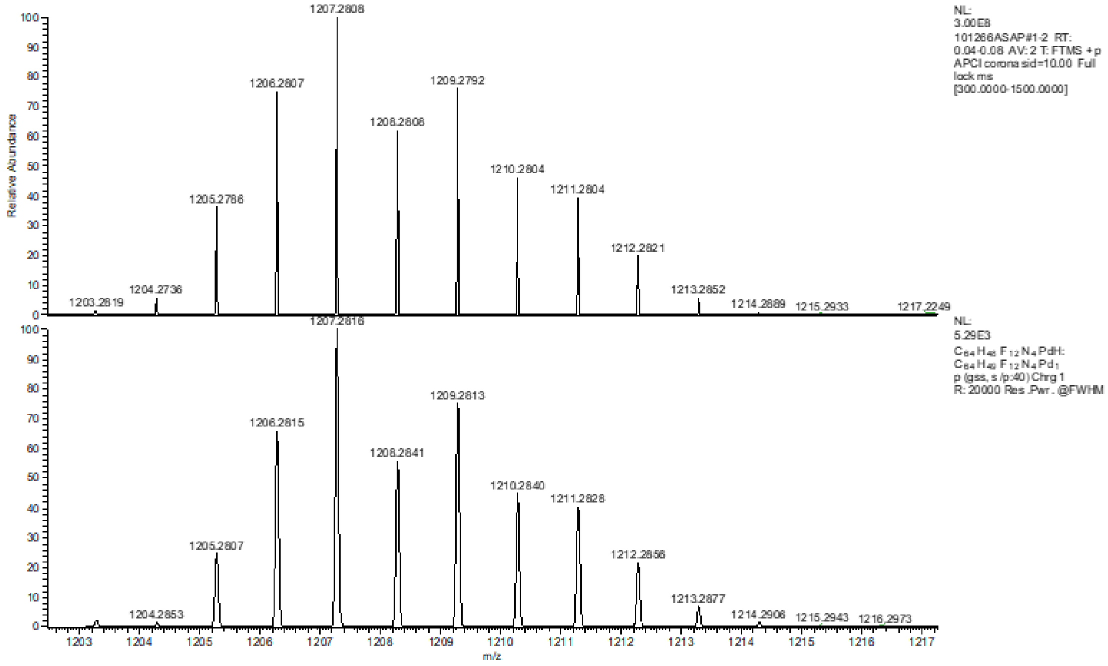

Figure S24 HRMS-ASAP(+) spectrum of **Pd4b**.

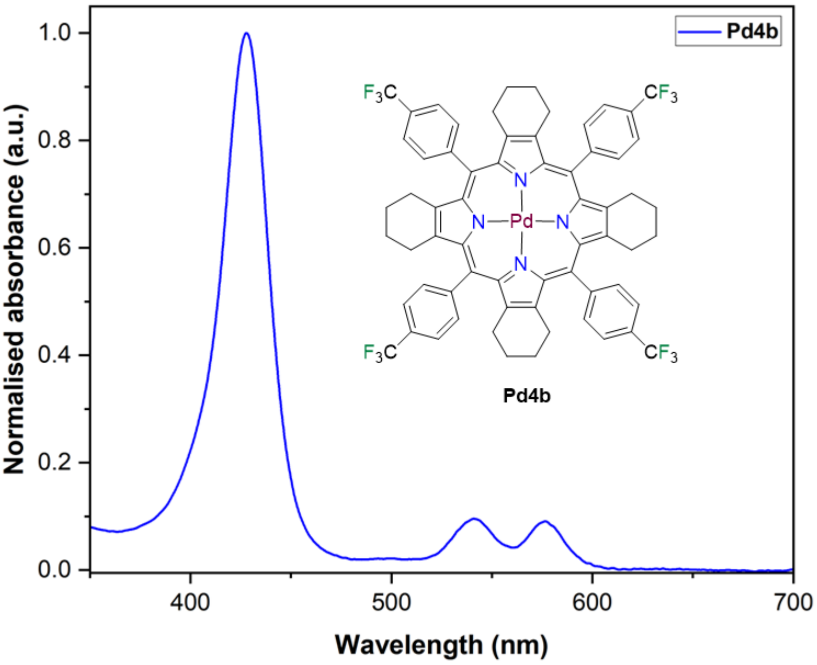

Figure S25. The UV-Vis electronic absorption spectrum of **Pd4b** in chloroform at a concentration of 2 μM.

4.4.8 Characterisation data of PtBP

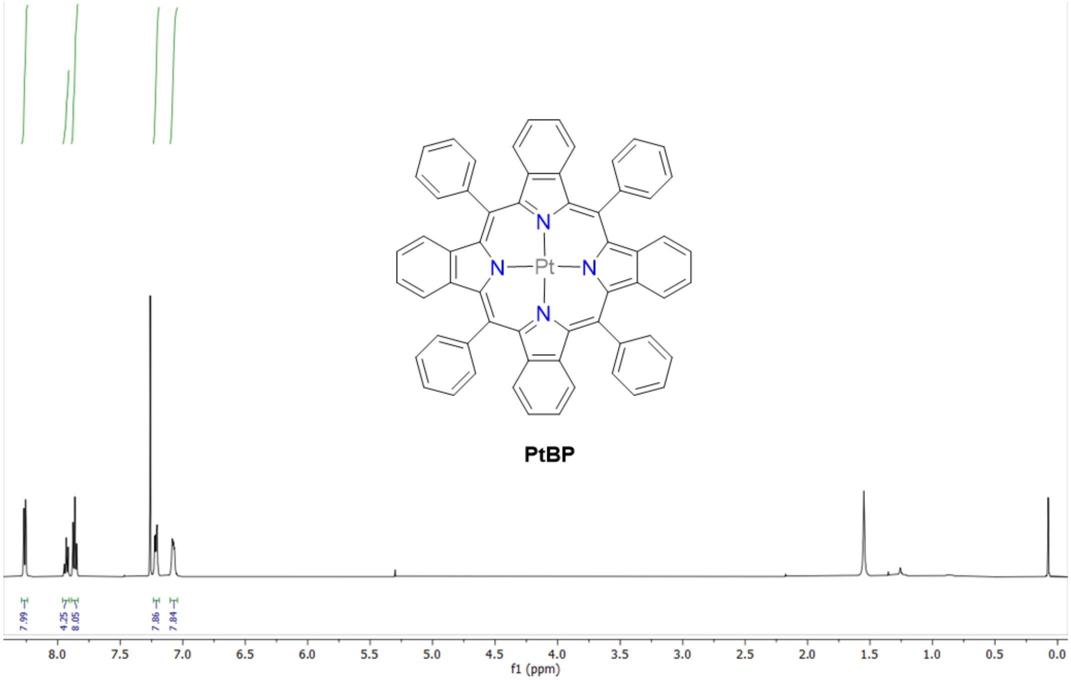

Figure S26. ^{1}H NMR spectrum of **PtBP** in $CDCl_3$.

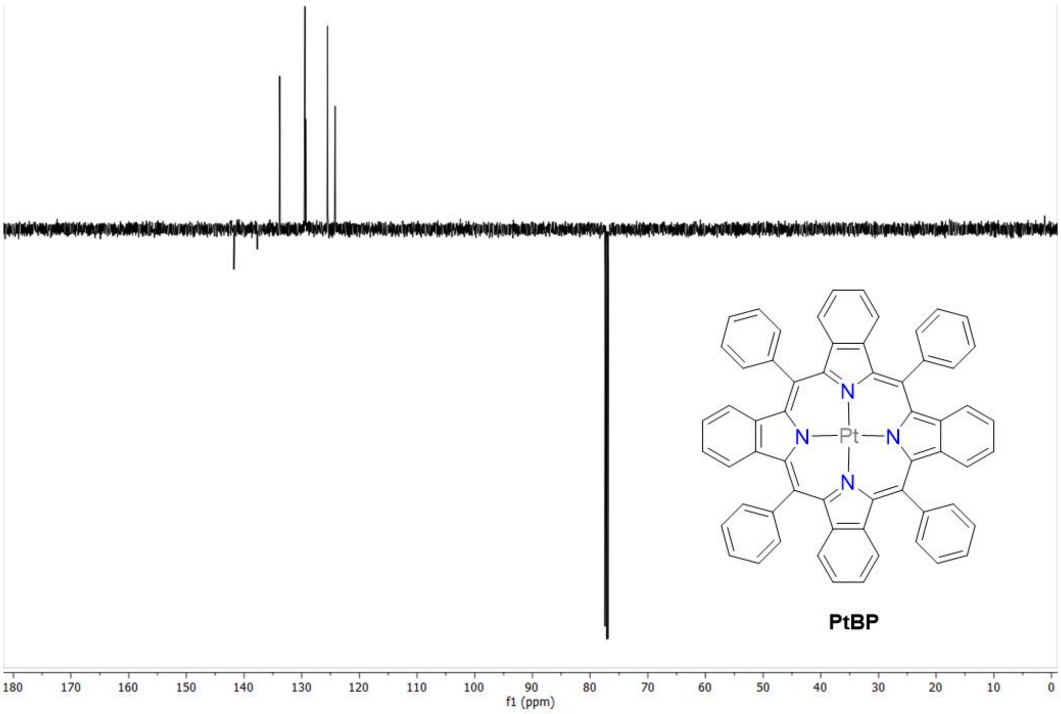

Figure S27. $^{13}C\{^{1}H\}$ DEPTQ NMR spectrum of **PtBP** in $CDCl_3$.

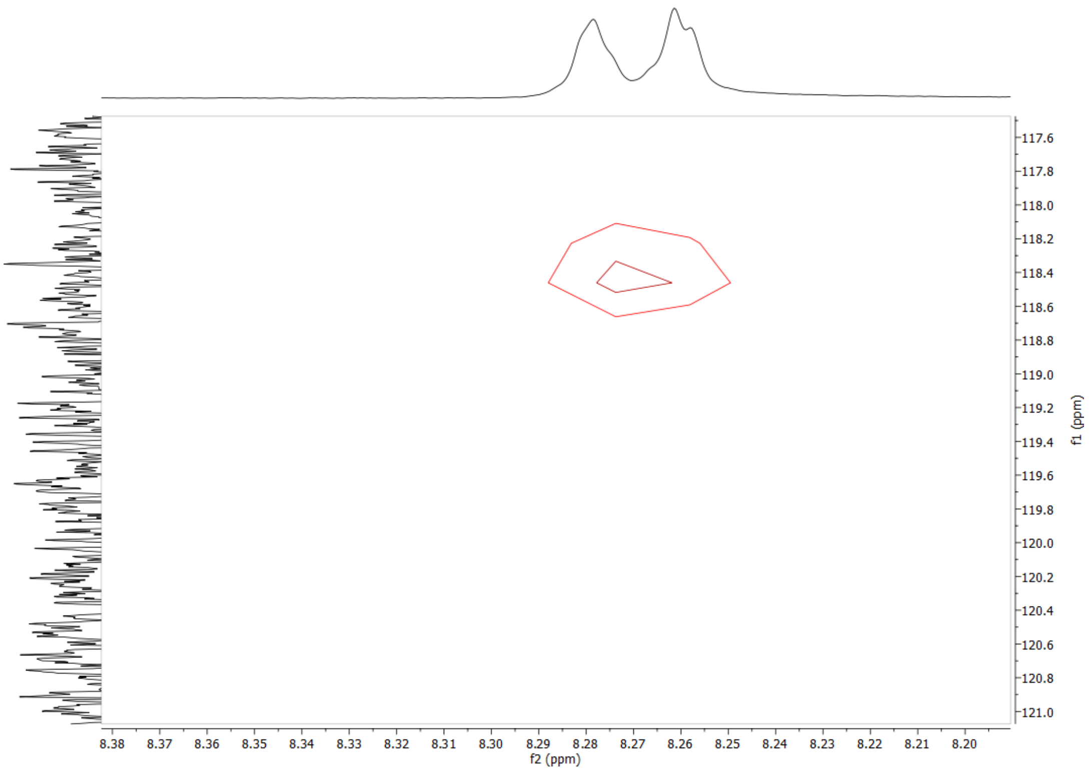

Figure S28. HMBC NMR spectrum of **PtBP** in $CDCl_3$, showing the *meso* ^{13}C signal coupling to the *ortho*-phenyl-H ^{1}H signal.

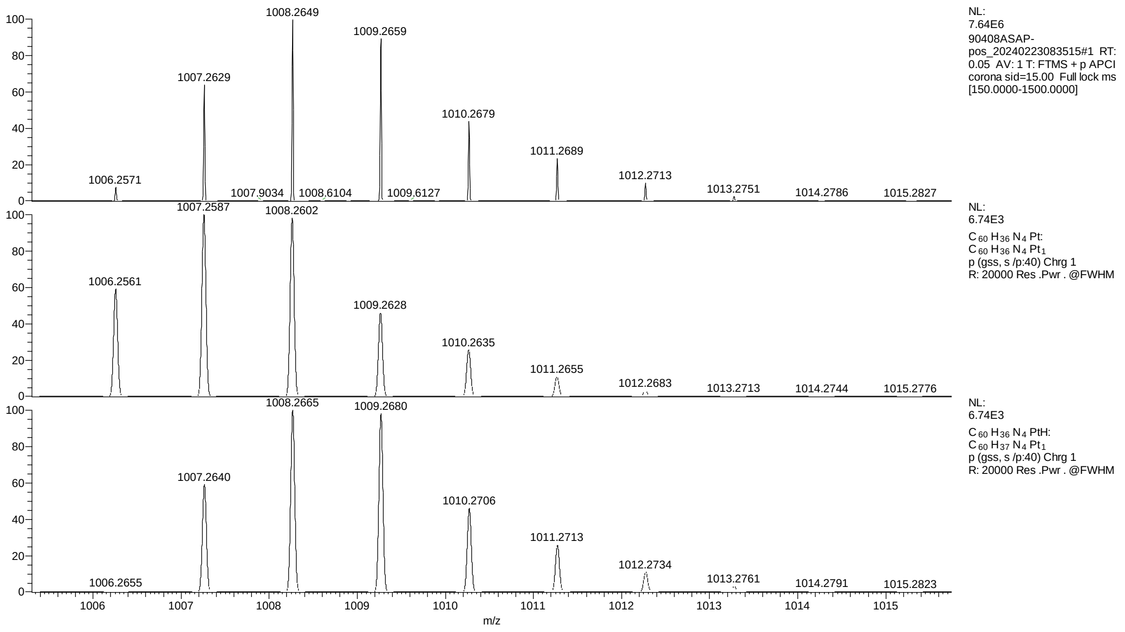

Figure S29. HRMS-ASAP(+) spectrum of **PtBP**.

4.4.9 Characterisation data of PdBP

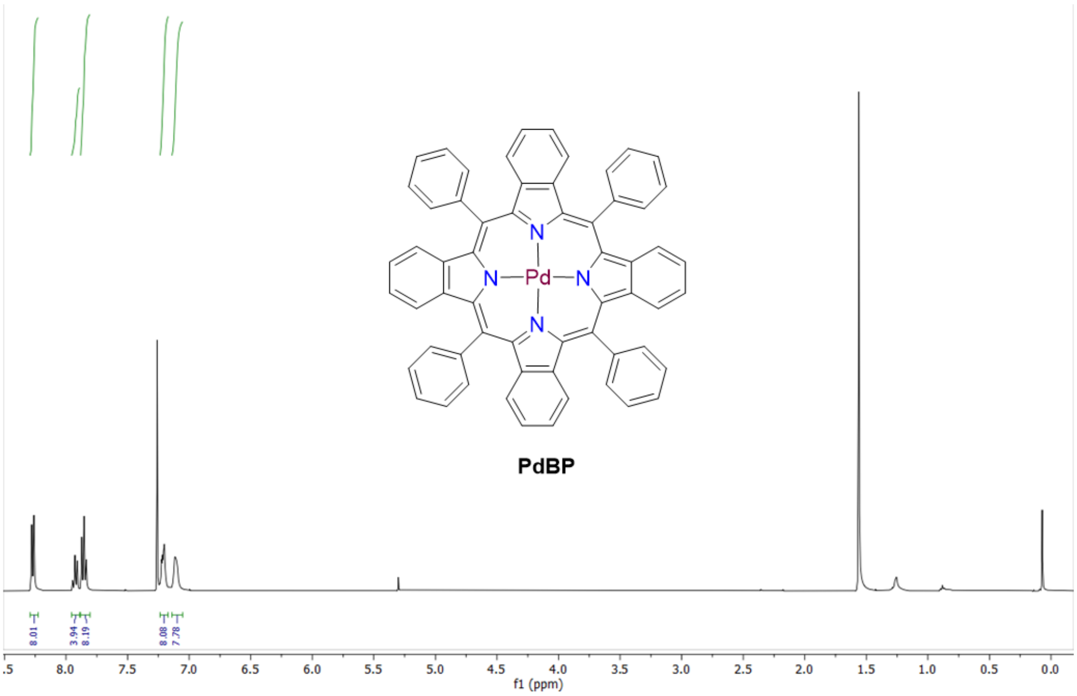

Figure S30. ^{1}H NMR spectrum of **PdBP** in $CDCl_3$.

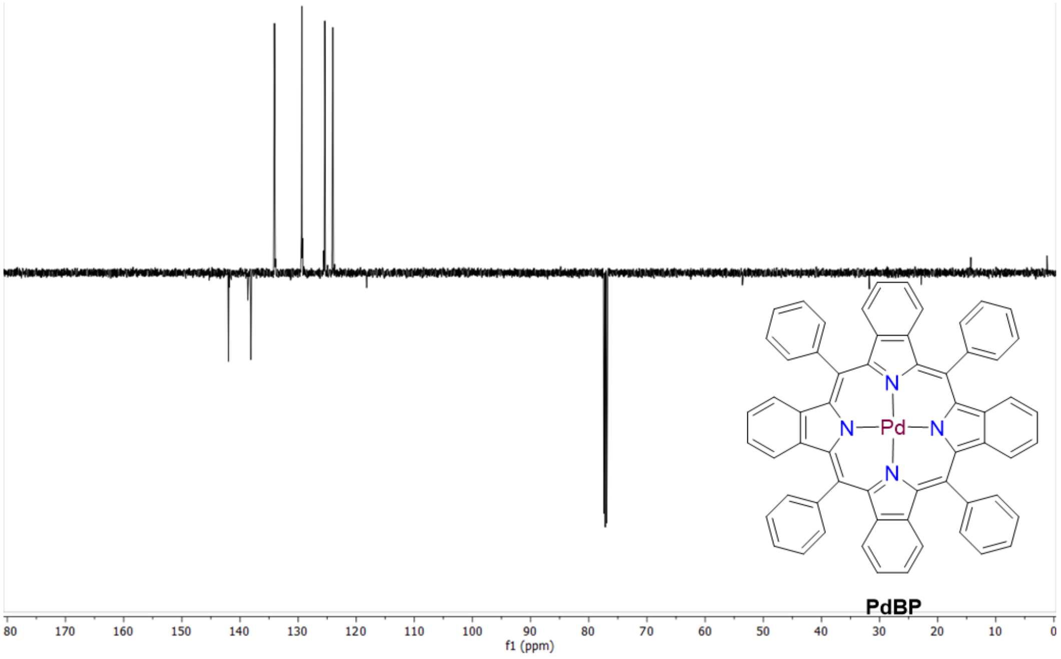

Figure S31. $^{13}C\{^{1}H\}$ DEPTQ NMR spectrum of **PdBP** in $CDCl_3$.

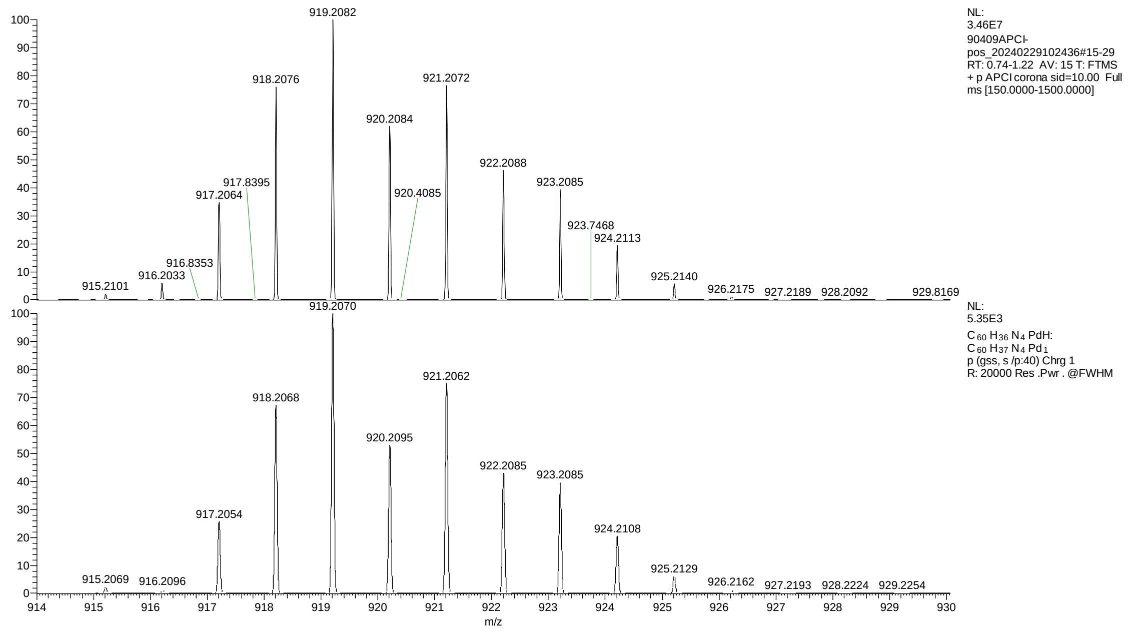

Figure S32. HRMS-ASAP(+) spectrum of **PdBP**.

4.4.10 Characterisation data of Pt-*p*CF_3-BP

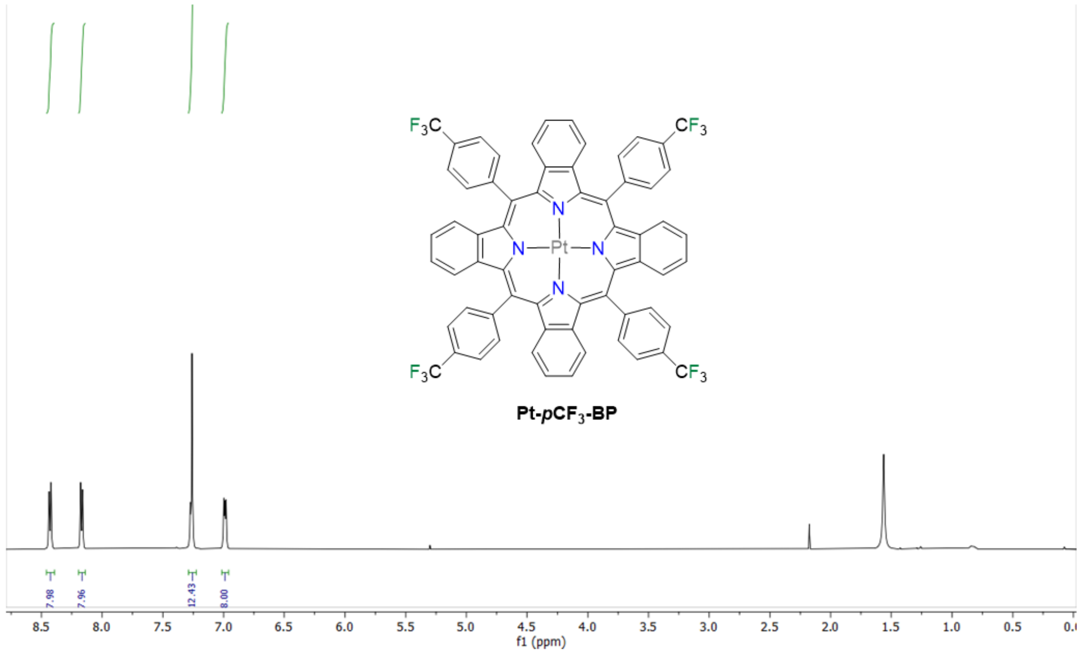

Figure S33. ^{1}H NMR spectrum of **Pt-*p*CF_3-BP** in $CDCl_3$.

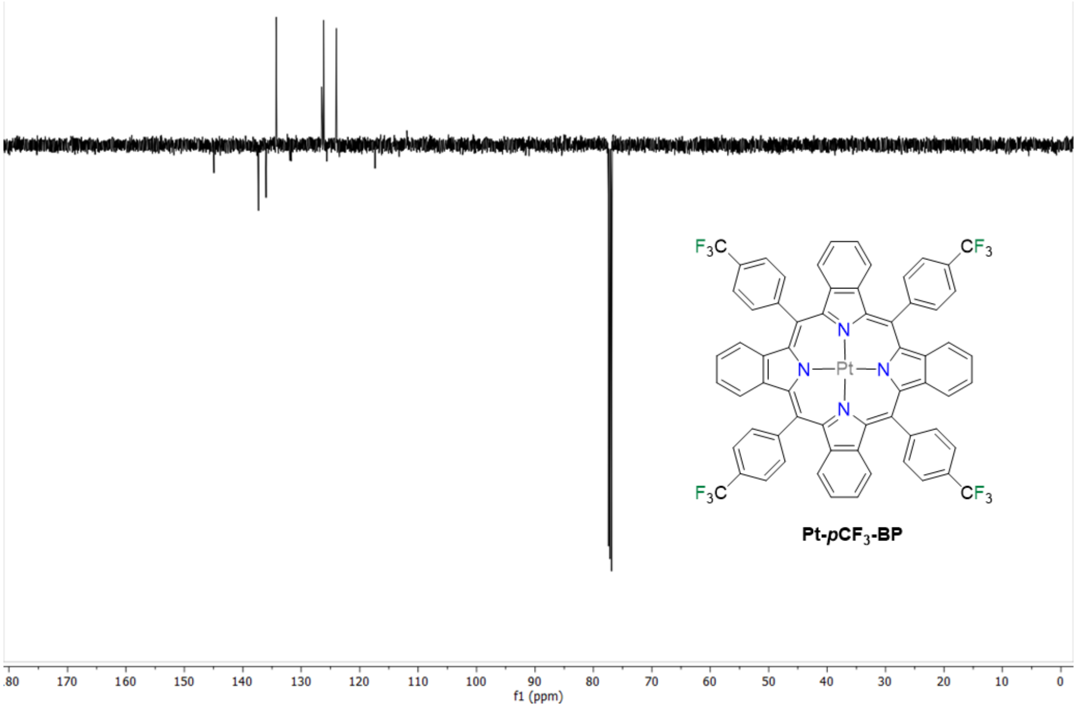

Figure S34. $^{13}C\{^{1}H\}$ DEPTQ NMR spectrum of **Pt-*p*CF$_3$-BP** in $CDCl_3$.

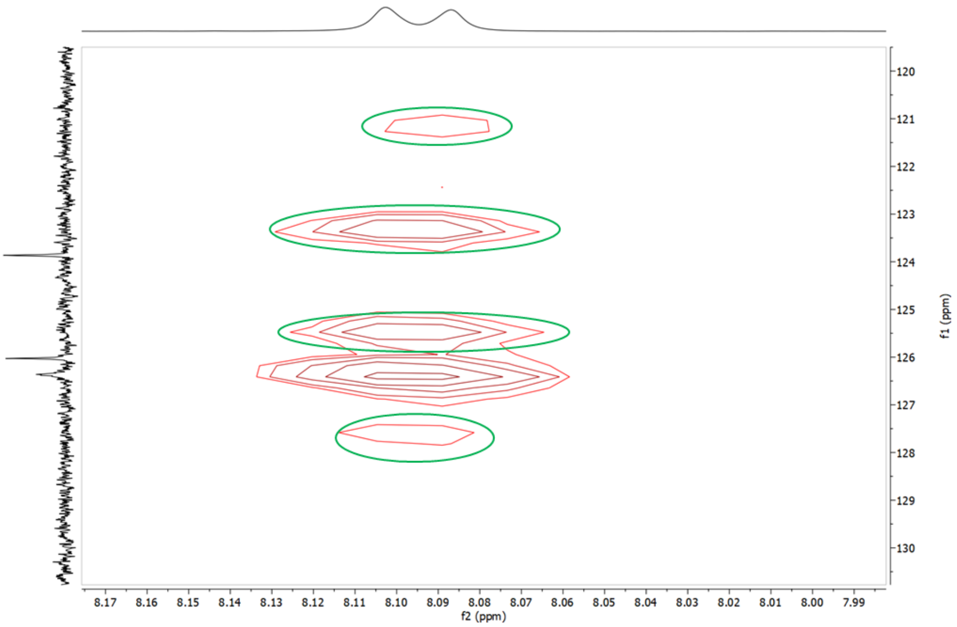

Figure S35. HMBC NMR spectrum of **Pt-*p*CF$_3$-BP** in $CDCl_3$, showing the quartet ($^{1}J_{C\text{-}F}$ = 271 Hz) CF_3 ^{13}C signal coupling to the *meta*-phenyl-H ^{1}H signal.

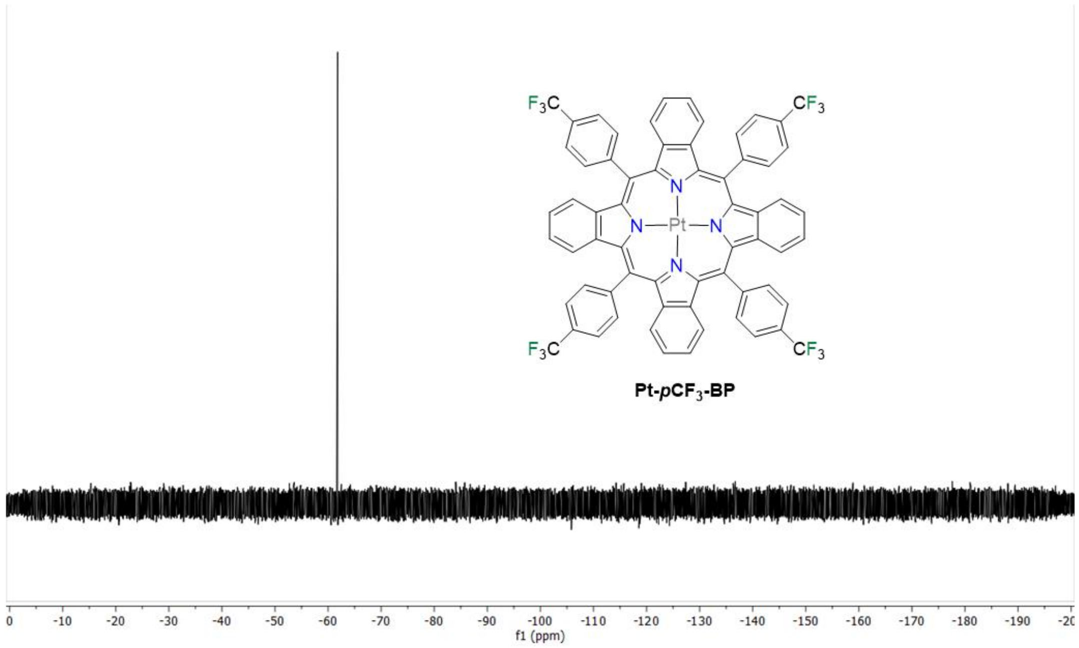

Figure S36. ^{19}F NMR of **Pt-*p*CF$_3$-BP** in $CDCl_3$.

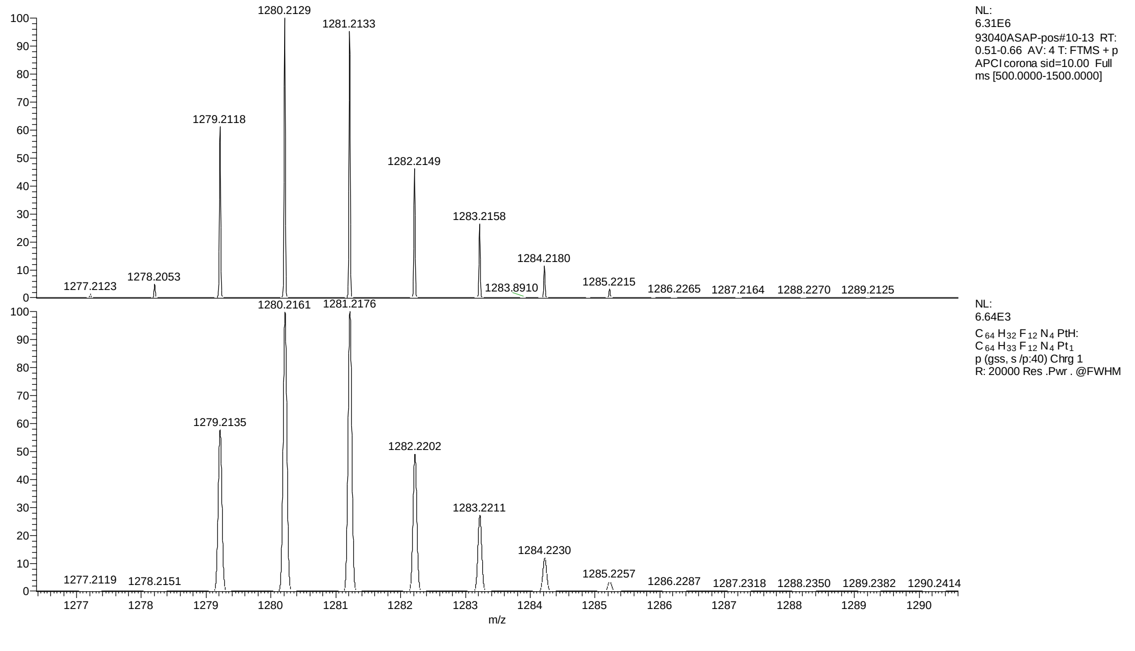

Figure S37. HRMS-ASAP(+) spectrum of **Pt-*p*CF$_3$-BP**.

4.4.11 Characterisation data of Pd-*p*CF_3-BP

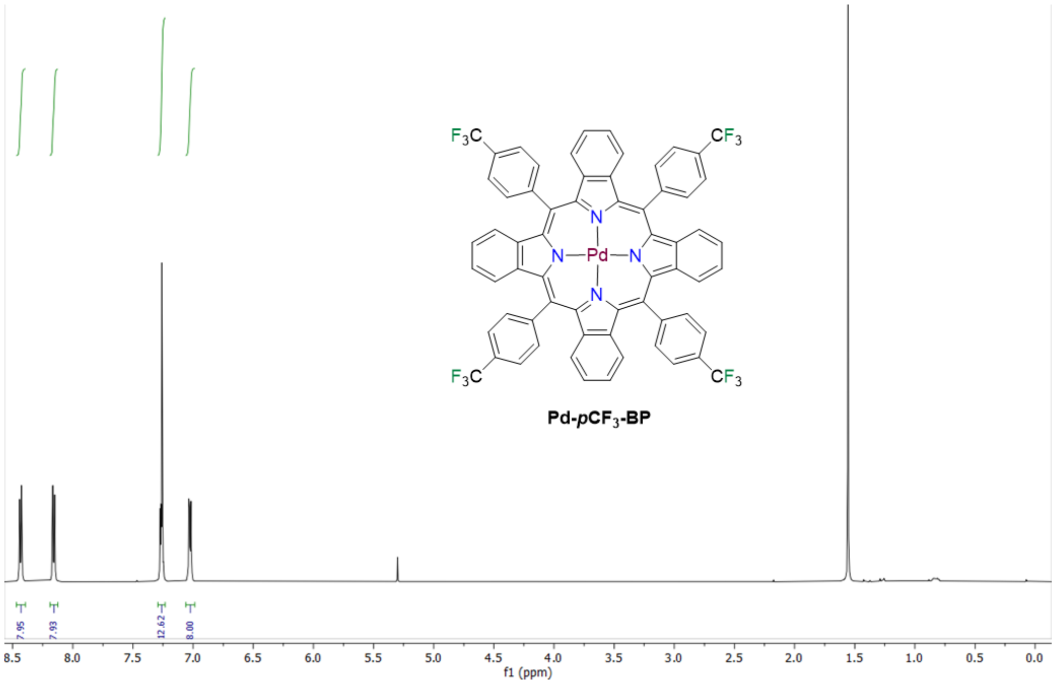

Figure S38 ^{1}H NMR spectrum of **Pd-*p*CF_3-BP** in $CDCl_3$.

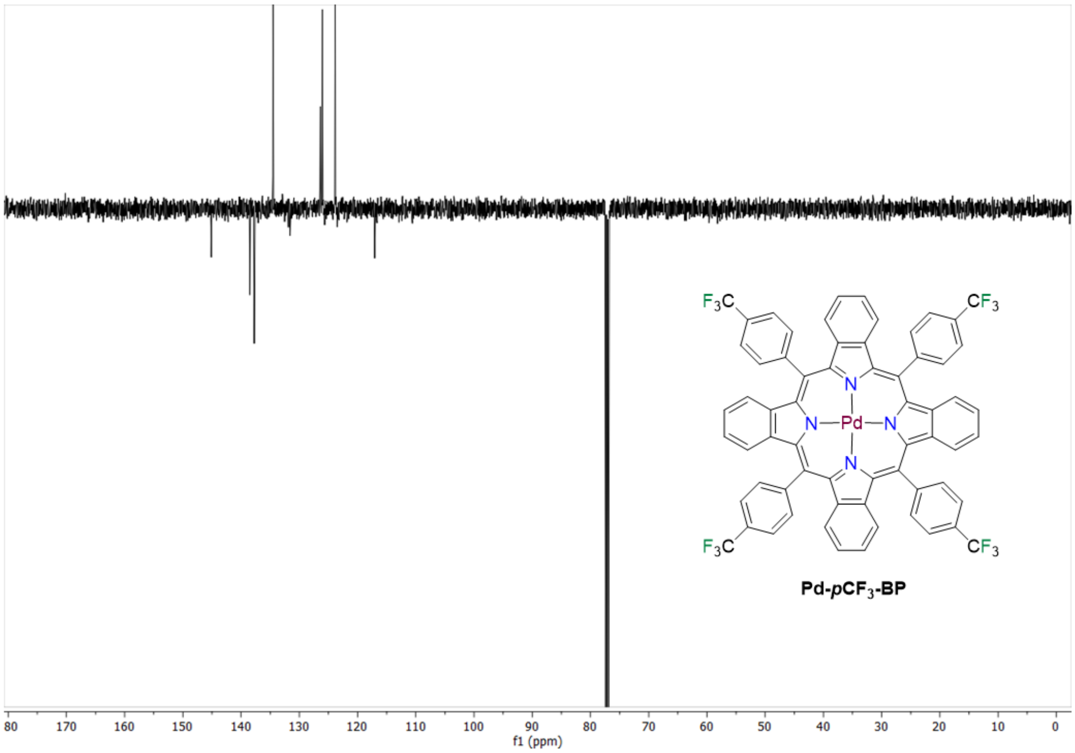

Figure S39. $^{13}C\{^{1}H\}$ DEPTQ NMR spectrum of **Pd-*p*CF_3-BP** in $CDCl_3$.

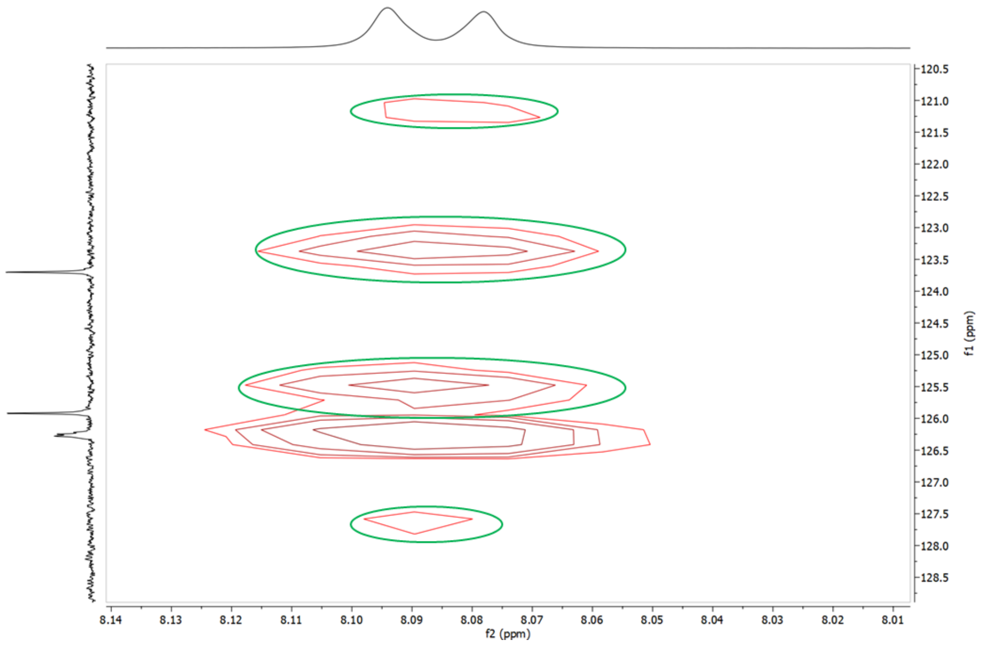

Figure S40. HMBC NMR spectrum of **Pd-*p*CF$_3$-BP** in CDCl$_3$, showing the quartet ($^1J_{C\text{-}F}$ = 270 Hz) CF$_3$ ^{13}C signal coupling to the *meta*-phenyl-H ^{1}H signal.

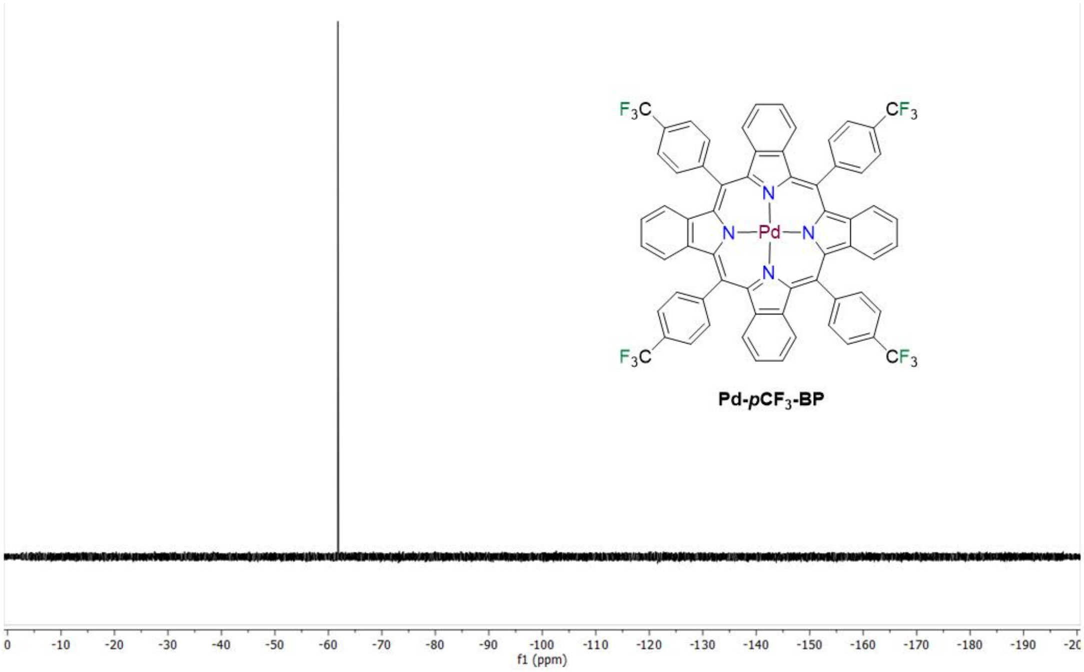

Figure S41. ^{19}F NMR spectrum of **Pd-*p*CF$_3$-BP** in CDCl$_3$.

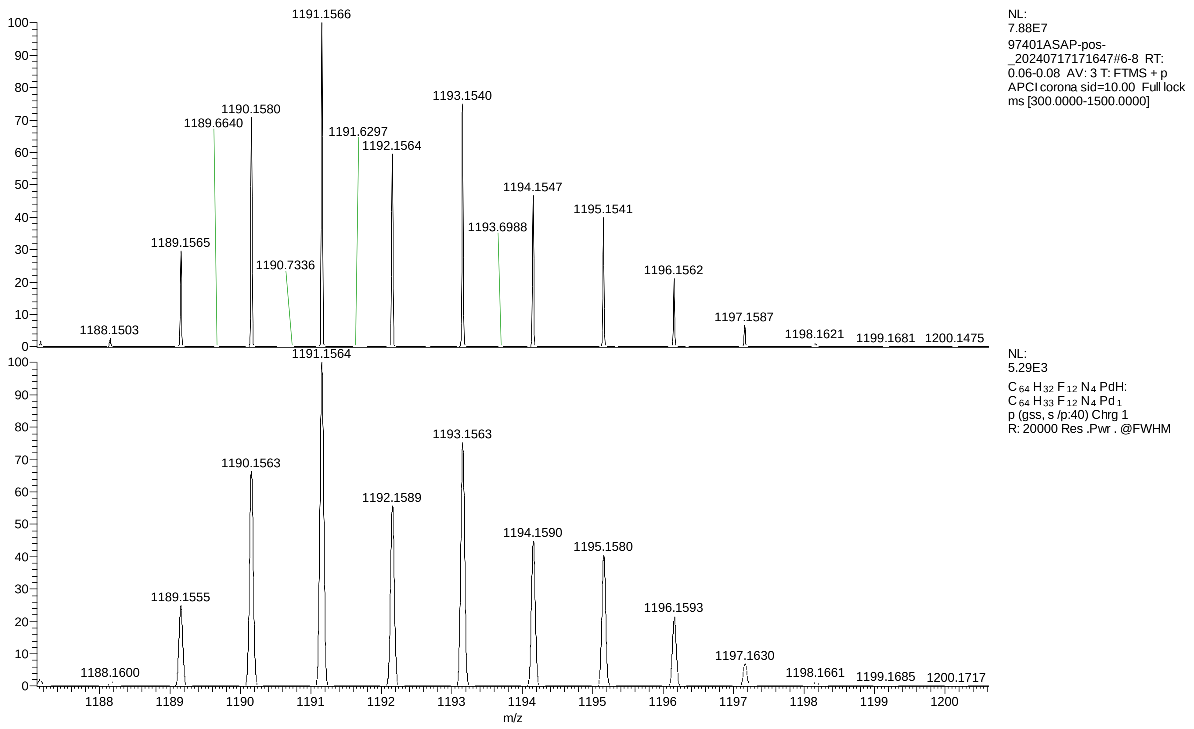

Figure S42. HRMS-ASAP(+) of **Pd-*p*CF_3-BP**.

5. UV-Vis electronic absorption spectroscopy

Table S1. Soret and Q Band maxima in nm, with the molar absorption coefficient (ε) in $M^{-1}cm^{-1}$, for the porphyrins in parenthesis. All values were recorded in chloroform.

Porphyrin	Soret band maximum (nm) [ε ($M^{-1}cm^{-1}$)]	Q band maxima (nm) [ε ($M^{-1}cm^{-1}$)]	
		Q_{abs}(1,0)	Q_{abs}(0,0)
**PtTFPP**[a]	392 [296,500]	507 [18,800]	540 [28,400]
**PtBP**	430 [210,600]	564 [17,300]	614 [123,200]
**Pt-*p*CF_3-BP**	427[216, 500]	567[17,800]	618[132,000]
**PdTFPP**[b]	407 [271,400]	519 [22,200]	552 [18,800]
**PdBP**	442[264,300]	577 [14,700]	629[102,800]
**Pd-*p*CF_3-BP**	440[278,600]	582[13,300]	631[114,130]

[a]Data previously published.[2]
[b]Data previously published.[4]

6. Lifetimes of Emission

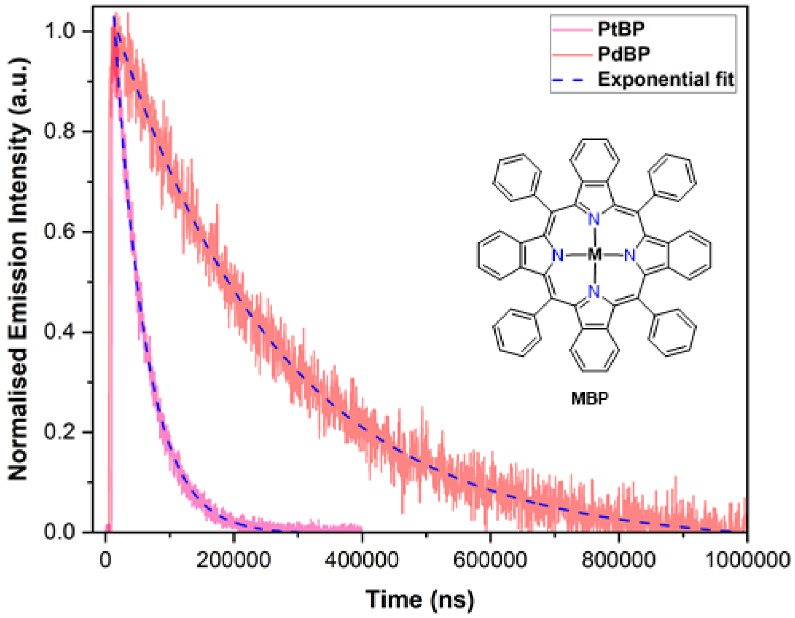

Figure S43. The lifetimes of emission in deoxygenated chloroform, $\tau_{(Ar)}$ at a concentration of 0.5 µM, fitted to mono-exponential decays for **PtBP** ($\tau_{(Ar)}$ = 48.7 µs, R^2 = 0.996) and **PdBP** ($\tau_{(Ar)}$ = 258.7 µs, R^2 = 0.984).

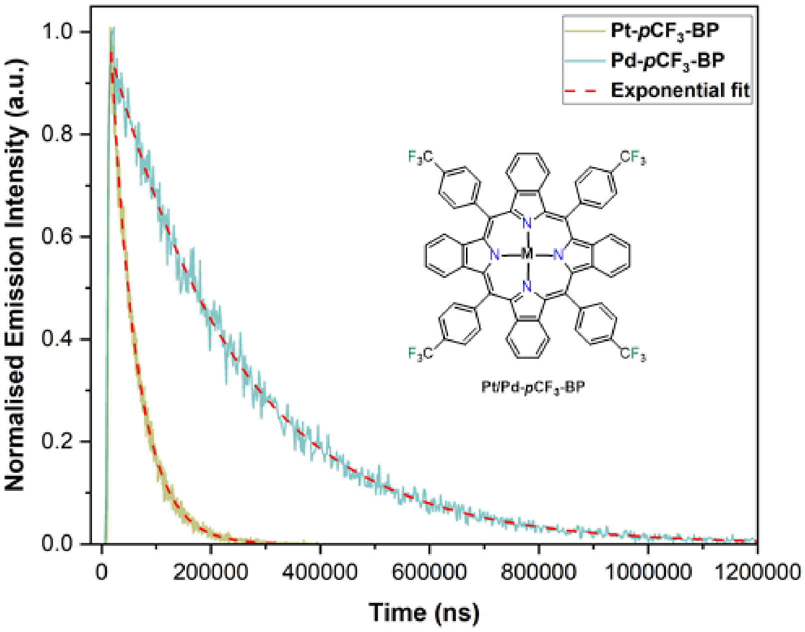

Figure S44. The lifetimes of emission in deoxygenated chloroform, $\tau_{(Ar)}$ at a concentration of 0.5 µM, fitted to mono-exponential decays for **Pt-*p*CF$_3$-BP** ($\tau_{(Ar)}$ = 47.5 µs, R^2 = 0.996) and **Pd-*p*CF$_3$-BP** ($\tau_{(Ar)}$ = 234.3 µs, R^2 = 0.994).

7. Emission Spectra

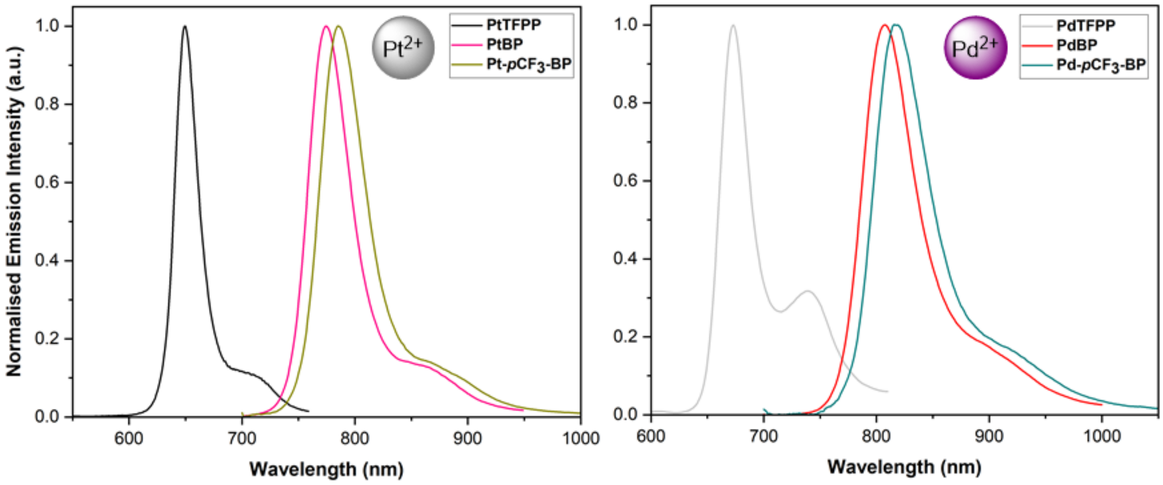

Figure S45. The normalised emission spectra of **Left) PtTFPP, PtBP** and **Pt-*p*CF$_3$-BP Right) PdTFPP, PdBP** and **Pd-*p*CF$_3$-BP**.

8. Polystyrene PSP performance studies

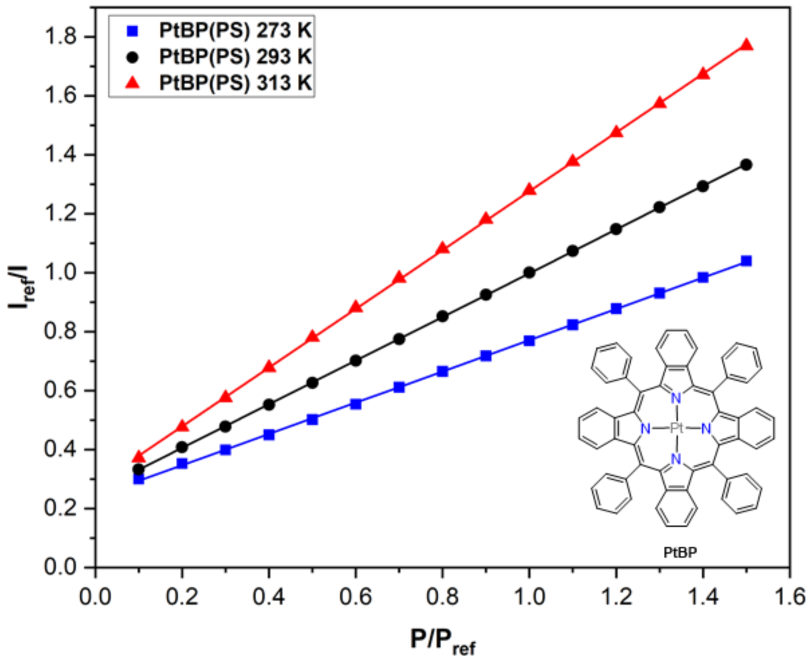

Figure S46. The modified Stern-Volmer calibrated luminescence response to pressure with associated linear fits of **PtBP(PS)** PSP at 273 K (R^2 = 1), 293 K (R^2 = 1) and 313 K (R^2 = 1). I_{ref} and P_{ref} are the luminescent intensity and pressure at 100 kPa and 293 K.

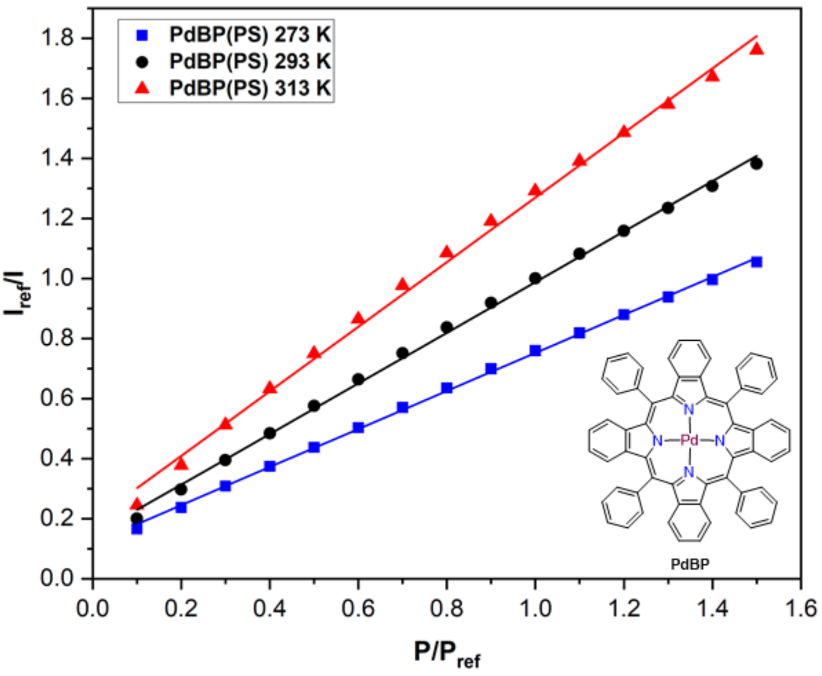

Figure S47. The modified Stern-Volmer calibrated luminescence response to pressure with associated linear fits of **PdBP(PS)** PSP at 273 K (R^2 = 0.999), 293 K (R^2 = 0.998) and 313 K (R^2 = 0.996). I_{ref} and P_{ref} are the luminescent intensity and pressure at 100 kPa and 293 K.

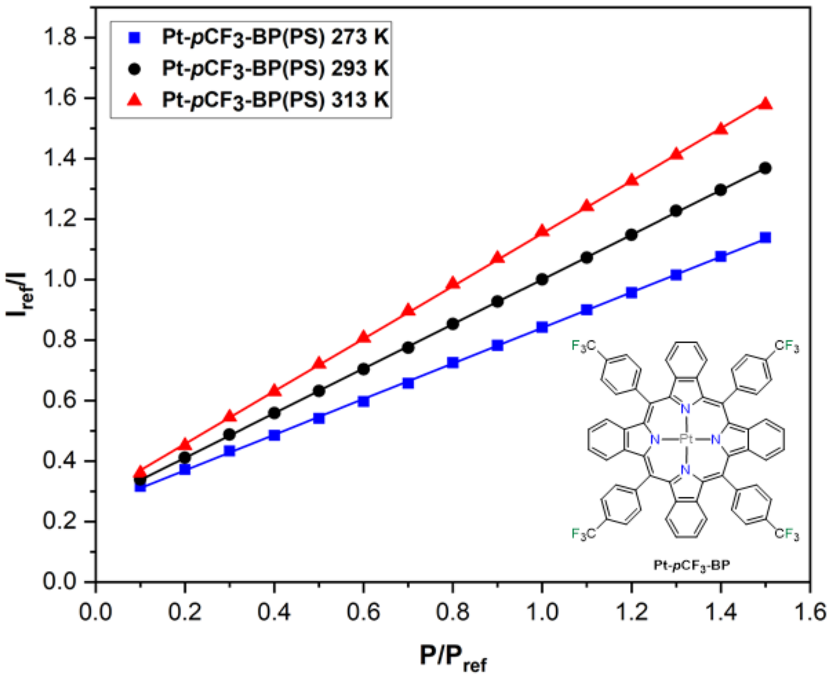

Figure S48. The modified Stern-Volmer calibrated luminescence response to pressure with associated linear fits of **Pt-*p*CF₃-BP(PS)** PSP at 273 K (R^2 = 1), 293 K (R^2 = 1) and 313 K (R^2 = 1). I_{ref} and P_{ref} are the luminescent intensity and pressure at 100 kPa and 293 K.

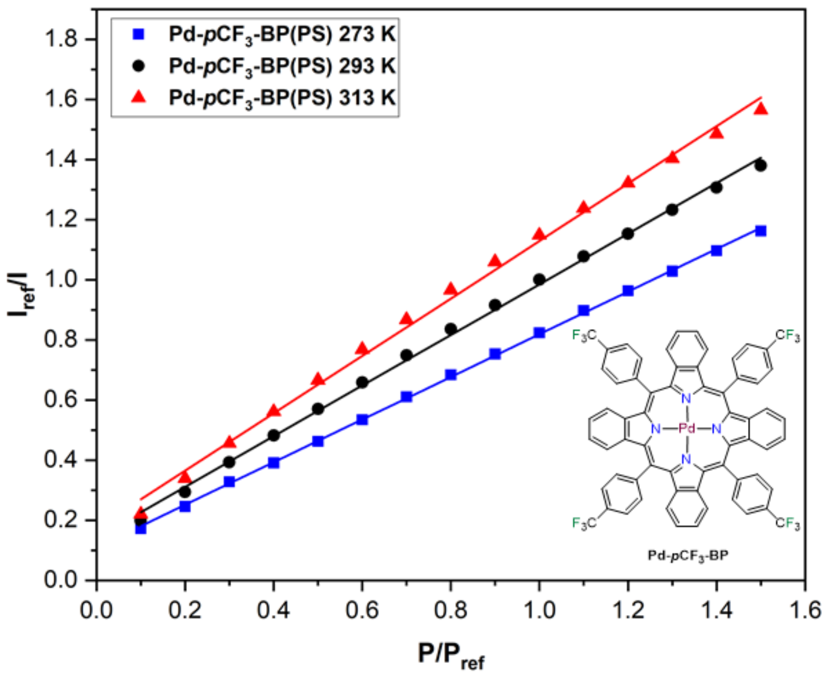

Figure S49. The modified Stern-Volmer calibrated luminescence response to pressure with associated linear fits of **Pd-*p*CF₃-BP(PS)** PSP at 273 K (R^2 = 1), 293 K (R^2 = 0.998) and 313 K (R^2 = 0.997). I_{ref} and P_{ref} are the luminescent intensity and pressure at 100 kPa and 293 K.

Table S2. The pressure sensitivity, S_p at 273, 293 and 313 K for the porphyrin polystyrene PSPs. The S_p is calculated using the linear form of the modified Stern-Volmer equation (equation S1).

Polystyrene-based PSP	Pressure sensitivity, S_p			Temperature sensitivity at 100 kPa S_T(100 kPa) (%/K)	Emission lifetime in argon saturated polystyrene $\tau_{(Ar)}$PS (µs)
	273 K	293 K	313 K		
**PtTFPP(PS)**[a]	0.448	0.662	0.970	1.56	55.3
**PtBP(PS)**	0.530	0.740	0.997	1.27	57.0
**Pt-*p*CF_3-BP(PS)**	0.588	0.737	0.870	0.79	56.3
**PdTFPP(PS)**[a]	0.624	0.913	1.228	1.71	891.4
**PdBP(PS)**	0.633	0.842	1.080	1.33	372.2
**Pd-*p*CF_3-BP(PS)**	0.709	0.842	0.955	0.81	353.4

[a]Data previously published.[4]

9. FIB PSP performance studies

Table S3. The pressure sensitivity, S_p at 273, 293 and 313 K for **Pt-*p*CF_3(0.8% w/w FIB)** . The S_p is calculated using the linear form of the modified Stern-Volmer equation (equation S1).

PSP	Pressure sensitivity, S_p			Temperature sensitivity at 100 kPa S_T(100 kPa) (%/K)
	273 K	293 K	313 K	
**Pt-*p*CF_3(0.8% w/w FIB)**	0.501	0.640	0.804	0.89

Table S4. The modified 2nd order Stern-Volmer (equation S2) constants for **Pt-*p*CF_3(3.2% w/w FIB)**, **Pd-*p*CF_3(3.2% w/w FIB)**, **Pd-*p*CF_3(3.2% w/w FIB)**.

PSP	*A*(T)			*B*(T)			Temperature sensitivity at 100 kPa S_T(100 kPa) (%/K)
	273K	293 K	313 K	273 K	293 K	313 K	
**Pt-*p*CF_3(3.2% w/w FIB)**	-0.26	-0.29	-0.33	0.84	0.98	1.10	0.44
**Pd-*p*CF_3(3.2% w/w FIB)**	-0.33	-0.39	-0.42	0.92	1.05	1.12	0.31
**Pd-*p*CF_3(0.8% w/w FIB)**	-0.30	-0.37	-0.44	0.93	1.09	1.22	0.60

10. References

1 M. Taniguchi, J. S. Lindsey, D. F. Bocian and D. Holten, *J. Photochem. Photobiol. C Photochem. Rev.*, 2021, **46**, 100401.

2 E. J. Nunn, D. Tsioumanis, G. F. S. Whitehead, T. B. Fisher, D. A. Roberts, M. K. Quinn and L. S. Natrajan, *Sens. Actuators B Chem.*, 2024, **409**, 135577.

3 30W Violet 430nm UV LED chip - 430nm UV LED - Lumixtar, https://www.lumixtar.com/30w-violet-430nm-high-power-led.html, (accessed 8 July 2024).

4 E. J. Nunn, D. Tsioumanis, G. F. S. Whitehead, T. B. Fisher, D. A. Roberts, M. K. Quinn and L. S. Natrajan, *ACS Omega*, 2024, **9**, 51580–51590.

5 O. S. Finikova, A. V. Cheprakov, I. P. Beletskaya, P. J. Carroll and S. A. Vinogradov, *J. Org. Chem.*, 2004, **69**, 522–535.
